# Determining the Optimal Dietary Barley-to-Corn Ratio for Arbas White Cashmere Goats: Insights from Rumen Fermentation, Meat Metabolomics, and Gastrointestinal Microbiota

**DOI:** 10.3390/ani16172800

**Published:** 2026-09-06

**Authors:** Lu Jin, Chunhua Zhang, Shengli Li, Chula Sa, Min Nuo, Ding Yang, Wenting Li, Le Fu, Panliang Chen, Yaxing Zhao, Bo Wang, Haizhou Sun

**Affiliations:** Inner Mongolia Academy of Agricultural and Animal Husbandry Sciences, Inner Mongolia Key Laboratory of Herbivores Nutrition, Key Laboratory of Grass-Feeding Livestock Healthy Breeding and Livestock Product Quality Control (Co-Construction by Ministry and Province), Ministry of Agriculture and Rural Affairs, Hohhot 010031, China; happyjinlu@163.com (L.J.); zhangch-602@163.com (C.Z.); lshl2000@163.com (S.L.); sachula17@163.com (C.S.); normanemailbox@163.com (M.N.); yangding@imaaahs.ac.cn (D.Y.); wuclwt@126.com (W.L.); fl1942068373@163.com (L.F.); 18148348926@163.com (Y.Z.);

**Keywords:** barley, cashmere goats, rumen microbiota, muscle metabolomics, n-3 polyunsaturated fatty acids

## Abstract

This study investigated the effects of graded dietary barley replacement for corn on growth performance, rumen fermentation, carcass traits, meat quality, fatty acid profiles, and the rumen microbiome in Arbas White Cashmere goats, and further applied untargeted muscle metabolomics to elucidate the metabolic mechanisms underlying meat quality alterations. Growth performance was monitored for all goats (n = 10 per group); rumen fermentation, carcass traits, serum biochemistry and meat quality were determined in six randomly selected goats per group (n = 6); and rumen microbiota profiling and muscle metabolomics were performed on three selected goats per group (n = 3). Partial barley substitution (33% and 67% of starch from barley) significantly improved final body weight and average daily gain (*p* < 0.05), whereas total replacement (100% barley starch) did not confer additional growth advantages. Moderate barley inclusion increased ruminal propionate concentration (*p* = 0.001) and enriched the fiber-degrading bacterium Prevotella, while total barley replacement markedly reduced rumen microbial diversity (Sobs, Chao1, ACE, and Shannon indices; *p* < 0.01) and depleted multiple fibrolytic and hydrogenotrophic genera, including *Christensenellaceae_R-7_group*, *NK4A214_group*, and *Methanobrevibacter*. The 33% barley group exhibited elevated serum total bile acids, FGF-19, and CYP27A1 levels (*p* < 0.05), suggestive of modulated bile acid metabolism, along with decreased glucose-6-phosphatase (*p* < 0.05), pointing to suppressed hepatic gluconeogenesis. Muscle metabolomics revealed that moderate barley substitution predominantly affected glycerophospholipid metabolism, with upregulation of PE(P-18:1/20:4) and PC(16:0/16:0), whereas total replacement induced extensive metabolic reprogramming characterized by downregulation of glycine and glutathione-related metabolites, indicative of compromised antioxidant defense. The 33% barley group also exhibited the highest muscular C18:3n-3 content (*p* < 0.01), while the 67% and total replacement groups showed increased C22:6n-3 deposition (*p* < 0.05). Correlation analysis confirmed that PE(P-18:1/20:4) and PC(16:0/16:0) were significantly positively correlated with ruminal propionate and ADG (r = 0.841–0.986; *p* < 0.05), and Prevotella abundance showed a strong negative trend with G-6-Pase (r = −0.771). Collectively, these findings demonstrate that replacing one-third of dietary corn starch with barley starch optimally balances productivity, rumen health, and meat quality through enrichment of Prevotella, enhanced propionate production, and modulation of bile acid metabolism, while excessive barley inclusion destabilizes the rumen ecosystem and triggers muscle oxidative stress, providing a theoretical basis for precision grain formulation in intensive cashmere goat production.

## Simple Summary

In intensive cashmere goat farming, corn serves as the main energy ingredient in diets, but persistently high corn prices have raised production costs for producers. Identifying cost-effective alternative grains while preserving animal growth, health and meat nutritional quality is of great practical value for the goat industry. This study explored the optimal replacement ratio of corn with barley in diets for Arbas White Cashmere goats. The results showed that replacing one-third of dietary corn with barley achieved the best overall effect: goats grew faster, maintained healthy digestive function in the rumen, and their muscle contained higher levels of health-promoting omega-3 fatty acids. In contrast, full replacement of corn with barley disrupted gut microbial balance and reduced muscle antioxidant capacity. These findings provide a practical feeding strategy to help farmers cut feed costs while ensuring meat quality and animal health.

## 1. Introduction

In ruminant systems, feed costs represent 60–70% of total production expenses, making the choice of energy ingredients crucial for profitability and performance [1]. Corn, valued for its high starch content and palatability, has been the primary energy source in intensive ruminant diets. However, persistently high corn prices, driven by market volatility, bioenergy competition, and regional supply–demand imbalances, have constrained profitability, spurring the search for cost-effective alternatives [2].

Barley, the fourth most important cereal globally, contains more crude protein and digestible fiber than corn and is rich in functional non-starch polysaccharides, notably β-glucans [3,4]. Although its high non-starch polysaccharide content limits use in monogastrics, the rumen microbiota efficiently degrades these carbohydrates into volatile fatty acids for energy [5]. Replacing corn with barley not only lowers feed costs but also alters rumen fermentation because barley starch degrades faster, shifting volatile fatty acid profiles and affecting ruminal pH, microbial protein synthesis, and energy efficiency [6,7]. However, excessive rumen-degradable starch may induce subacute acidosis, compromising animal health and productivity [8]. Determining the optimal barley inclusion level is therefore critical for ruminant performance.

Cashmere goats are economically important small ruminants in northern China [9]. Arbas White Cashmere goats, a dual-purpose breed adapted to arid environments, have muscle rich in functional fatty acids [10]. With production shifting from grazing to confinement, high-concentrate diets are increasingly common. However, studies on the effects of barley replacement on rumen fermentation, carcass traits, meat quality, and fatty acid composition in cashmere goats remain limited, with no systematic evaluation of graded inclusion levels. Moreover, the modulatory effects on serum bile acid metabolism, which serves as a key regulator of lipid digestion, are unknown [11].

Advances in 16S rRNA gene sequencing and untargeted metabolomics now enable comprehensive analysis of diet–microbe–host interactions. 16S sequencing reveals gut microbiota composition and identifies dietary biomarkers, providing mechanistic insight into how diet influences gut health [12]. Untargeted metabolomics can systematically profile muscle metabolite changes and, through pathway enrichment, elucidate the metabolic basis of meat quality variation [13]. However, single-omics approaches capture only one dimension of the regulatory process: ruminal microbiota profiling alone cannot explain how microbial changes translate to host muscle phenotypes, whereas muscle metabolomics alone cannot identify upstream microbial drivers. Integrating rumen microbiota with muscle metabolomics makes it possible to trace the entire regulatory cascade from dietary grain manipulation, through ruminal microbial remodeling and fermentative shifts, to downstream alterations in host systemic metabolism and intramuscular metabolite profiles, thus providing a more holistic and mechanistic understanding of how cereal sources shape meat quality. However, no study has integrated these approaches to examine how barley replacement simultaneously affects the gastrointestinal microbiota and muscle metabolome in cashmere goats.

We hypothesized that moderate barley substitution, replacing one-third to two-thirds of dietary corn starch, would improve growth performance, optimize rumen fermentation, and enhance meat nutritional quality by enriching fibrolytic bacteria and increasing propionate production, and that these beneficial effects would be accompanied by altered serum bile acid profiles and coordinated changes in muscle glycerophospholipid metabolism, thereby linking ruminal microbial activity to host systemic metabolic responses. In contrast, we expected that full replacement of corn with barley would disrupt rumen microbial homeostasis, reduce microbial diversity, and induce oxidative stress-related metabolic disturbances in muscle, thereby negating the beneficial effects on growth performance and meat quality. To test these hypotheses, we combined a feeding trial with muscle metabolomics and 16S rRNA sequencing to investigate the effects of graded barley replacement on rumen fermentation, carcass traits, meat quality, serum bile acid indices, muscle fatty acid profiles, and rumen microbial community structure, and to identify differential muscle metabolites and key pathways. The objective was to determine the optimal barley replacement level for Arbas White Cashmere goats, providing a foundation for precision nutrition and cost-efficient production.

## 2. Materials and Methods

### 2.1. Animals, Experimental Design, and Diets

Forty healthy, castrated male Albas White Cashmere goats (approximately 4 months of age, initial body weight 15.0 ± 1.0 kg) were selected for the experiment. Goats were first stratified by initial body weight into blocks, and then randomly assigned within each block to four dietary treatment groups (n = 10 per group) in a completely randomized design using a random number generator. The sample size of 10 goats per group for growth performance monitoring conforms to standard power requirements for ruminant feeding trials, providing ≥80% statistical power to detect a 10% difference in average daily gain at the *p* < 0.05 significance level. Six goats per group were randomly selected for slaughter and routine phenotypic assays, which is a widely adopted sample size for carcass and meat quality studies in small ruminants, balancing statistical reliability, experimental cost and animal welfare. Three goats per group were further selected for exploratory omics profiling, which is common for hypothesis-generating multi-omics studies; however, the relatively small sample size for omics analyses means that findings should be interpreted as preliminary and require further validation in larger cohorts. All 10 goats per group were used for growth performance monitoring to ensure sufficient statistical power for production trait analysis and to reserve spare animals for unexpected events (e.g., illness or injury) during the feeding trial. At the end of the trial, six goats per group were randomly selected from the 10 animals for slaughter and subsequent sample collection (blood, rumen fluid, carcass and meat quality analyses); three goats were further randomly selected from the six slaughtered animals for untargeted metabolomics and rumen microbiota profiling. The four dietary treatments were CS (100% corn starch), 33BS (33% barley starch + 67% corn starch), 67BS (67% barley starch + 33% corn starch), and 100BS (100% barley starch). The ingredient composition and nutrient levels of the basal diet are presented in Table 1. The experiment lasted for 60 days, consisting of a 15-day adaptation period followed by a 45-day formal feeding trial.

During the trial, all goats were fed a total mixed ration (TMR) at 5% of body weight daily, offered in two equal meals. Clean water was available ad libitum. Housing conditions and management practices were kept uniform, and feed intake was recorded daily by designated personnel.

### 2.2. Growth Performance

Feed refusals were collected and weighed daily before the morning feeding to determine actual feed intake on a dry matter basis. Average daily feed intake (ADFI) was calculated as the difference between daily feed offer and daily refusal, adjusted for the dry matter content of the diet, and then averaged over each measurement period. Body weight was measured before the morning feeding on the same schedule. Average daily feed intake (ADFI), average daily gain (ADG), and feed-to-gain ratio (F/G) were calculated for each 10-day period and for the entire 45-day trial.

### 2.3. Carcass Traits and Meat Quality

At the end of the feeding trial, goats were approximately 6 months of age. All selected goats were subjected to a 12-h feed withdrawal with free access to water prior to slaughter. Slaughter was performed in a certified commercial abattoir following standard humane slaughter procedures approved by the Institutional Animal Care and Use Committee: goats were stunned by captive bolt pistol, immediately exsanguinated, and then processed according to conventional meat industry protocols. Six randomly selected goats per group (from the initial 10 animals) were used for carcass measurements. Carcass traits, including pre-slaughter live weight, carcass weight, dressing percentage, net meat weight, bone-to-meat ratio, GR value, and loin eye area, were determined following standard procedures [14]. For meat quality assessment, *Longissimus dorsi* samples were taken between the 12th and 13th ribs. Measurements included pH at 45 min and 24 h postmortem, cooking loss, drip loss, and Warner–Bratzler shear force, as described by [15].

### 2.4. Sample Collection

#### 2.4.1. Rumen Fluid

At the end of the trial, six goats per group (the same animals used for carcass analysis) were slaughtered, and rumen fluid samples were collected. A portion of the fluid was immediately used for pH measurement. Another portion was filtered through four layers of cheesecloth, and the supernatant was aliquoted into 5 mL tubes and stored at −20 °C for ammonia nitrogen and volatile fatty acid (VFA) analysis. An additional aliquot was placed in 5 mL cryogenic vials and stored at −80 °C for ruminal microbiota profiling.

#### 2.4.2. Blood Samples

At slaughter, blood samples (10 mL) were collected from the jugular vein of six goats per group (the same slaughtered animals) into vacuum tubes without anticoagulant. Serum was obtained by centrifugation at 3000× *g* for 15 min at 4 °C, aliquoted into 1.5 mL Eppendorf tubes, and stored at −20 °C.

#### 2.4.3. Muscle Samples

From six goats per group (the same slaughtered animals), two portions of the *Longissimus dorsi* (approximately 250 g each) were collected after removing visible fascia and stored at −20 °C. A section of the rib cut (approximately 500 g) was also obtained and stored at −20 °C for meat quality assessments.

### 2.5. Serum Biochemical Parameter Determination

Fasting blood samples were collected from the jugular vein of six goats per group at the end of the feeding trial. Blood samples (10 mL) were drawn into vacuum tubes without anticoagulant and centrifuged at 3000× *g* for 15 min at 4 °C to obtain serum. Serum concentrations of glucose (GLU) and total bile acids (TBA) were measured using an automated biochemical analyzer (Hitachi 7020, Hitachi, Tokyo, Japan) with commercial assay kits. Serum concentrations of fibroblast growth factor 15 (FGF15), fibroblast growth factor 19 (FGF-19), fibroblast growth factor 21 (FGF-21), cholesterol 7α-hydroxylase (CYP7A1), sterol 27-hydroxylase (CYP27A1), bile salt hydrolase (BSH), fructose-1,6-bisphosphatase 1 (FBP1), phosphoenolpyruvate carboxykinase (PEPCK), glucose-6-phosphatase (G-6-Pase), oxysterol 7α-hydroxylase (CYP7B1), glucagon-like peptide 1 (GLP-1), and insulin (INS) were determined using commercially available sheep-specific enzyme-linked immunosorbent assay (ELISA) kits. All ELISA procedures were performed in strict accordance with the manufacturers’ protocols. All ELISA kits used in this study have been validated for cross-reactivity with caprine serum samples by the manufacturer, with reported cross-reactivity rates > 90% for the target analytes in goats. The kit performance (parallelism, recovery rate, and intra-/inter-assay coefficients of variation) has been verified in previous ruminant studies, and all assays were performed in strict accordance with the manufacturers’ protocols. Absorbance values were read using a microplate reader, and sample concentrations were calculated from the corresponding standard curves. All assays were conducted by Beijing Zhongtong Lanbo Medical Laboratory Co., Ltd. (Beijing, China).

### 2.6. Rumen Fermentation Parameters

Rumen fluid pH was measured immediately after collection using a portable pH meter. Ammonia nitrogen concentration was determined by the colorimetric phenol-hypochlorite method. VFA concentrations were analyzed using an Agilent 7890B gas chromatograph (Agilent Technologies, Santa Clara, CA, USA) equipped with a flame ionization detector (FID) and an HP-INNOWax capillary column (30 m × 0.25 mm × 0.25 μm; Agilent Technologies). The oven temperature program was set as follows: initial temperature 80 °C, held for 1 min, increased to 120 °C at 10 °C/min, held for 3 min, then increased to 180 °C at 20 °C/min, and held for 2 min. The injector and detector temperatures were set at 220 °C and 250 °C, respectively. Nitrogen was used as the carrier gas at a flow rate of 1.0 mL/min. The split ratio was 20:1, and the injection volume was 1 μL. 2-Ethylbutyric acid was used as the internal standard for quantification [16].

### 2.7. Fatty Acid Composition Analysis

Total lipids were extracted from *Longissimus dorsi* samples, and fatty acid methyl esters (FAMEs) were prepared according to the method of O’Fallon et al. [17]. FAMEs were separated and quantified using an Agilent 7890B gas chromatograph equipped with a flame ionization detector and an SP-2560 capillary column (100 m × 0.25 mm × 0.20 μm; Supelco, Bellefonte, PA, USA). The oven temperature program was: initial temperature 100 °C, held for 5 min, increased to 180 °C at 4 °C/min, held for 10 min, then increased to 240 °C at 3 °C/min, and held for 15 min. The injector temperature was set at 250 °C and the detector temperature at 260 °C. Hydrogen was used as the carrier gas at a constant flow rate of 1.2 mL/min. The split ratio was 50:1, and the injection volume was 1 μL. Individual fatty acids were identified by comparison with a 37-component FAME standard mixture and expressed as a percentage of total identified fatty acids.

### 2.8. Untargeted Metabolomics of Longissimus dorsi

Three goats per group were randomly selected from the six slaughtered animals for untargeted metabolomic profiling. It should be noted that a sample size of three biological replicates per group provides only exploratory statistical power and is insufficient for robust population-level inference. The metabolomics findings are intended for hypothesis generation and identification of candidate metabolic pathways, and key differentially abundant metabolites and enriched pathways should be interpreted with caution and validated in larger independent cohorts. Briefly, 50 mg of tissue was extracted with 800 μL of pre-chilled methanol–water (1:1, *v*/*v*) containing 2-chlorophenylalanine as an internal standard. Following homogenization, incubation at −20 °C, and centrifugation, the supernatant was dried and reconstituted in acetonitrile–water (1:1, *v*/*v*) for analysis. A pooled quality control (QC) sample was prepared by mixing equal aliquots from all sample extracts and injected every 10 experimental samples to monitor instrumental stability. Only metabolites with a relative standard deviation (RSD) of peak area < 30% across all QC injections were retained for downstream statistical analysis [18].

Metabolite profiling was conducted on a Vanquish Horizon UHPLC system coupled to a Q Exactive HF-X mass spectrometer (Thermo Scientific, Waltham, MA, USA) with an ACQUITY UPLC HSS T3 column (2.1 × 100 mm, 1.8 μm; Waters, Milford, MA, USA). Raw data were acquired in positive (POS) and negative (NEG) electrospray ionization modes [19]. Peak detection, peak area normalization by internal standard, and probabilistic quotient normalization (PQN) were performed using Progenesis QI (v2.3; Waters). All samples were extracted and analyzed in a single analytical batch to eliminate between-batch variation; intra-batch signal drift was corrected based on the pooled QC samples [20].

Metabolites were identified by matching accurate mass (mass error < 10 ppm) and MS/MS fragmentation spectra against HMDB (v4.0) and METLIN databases [20]. A metabolite was considered positively annotated when at least three characteristic fragment ions were matched and the spectral similarity score exceeded 0.7.

### 2.9. Rumen 16S rRNA Gene Amplicon Sequencing and Analysis

Rumen fluid samples from three goats per group (the same animals used for metabolomics analysis) were used for microbiota profiling. Consistent with the metabolomics analysis, a sample size of three biological replicates per group for 16S rRNA gene sequencing is exploratory in nature and has limited statistical power for detecting low-abundance and rare taxa. The analysis focuses on overall community structure and dominant biomarker taxa, and conclusions regarding low-abundance genera should be interpreted with appropriate caution. Microbial genomic DNA was extracted using the E.Z.N.A.^®^ Soil DNA Kit (Omega Bio-tek, Norcross, GA, USA) following the manufacturer’s instructions. DNA concentration and purity were assessed with a NanoDrop 2000 spectrophotometer (Thermo Scientific) and agarose gel electrophoresis. All samples exhibited A260/A280 ratios between 1.8 and 2.0 and were of sufficient quality for library preparation. The V3–V4 hypervariable region of the 16S rRNA gene was amplified with the universal primers 338F (5′-ACTCCTACGGGAGGCAGCAG-3′) and 806R (5′-GGACTACHVGGGTWTCTAAT-3′) [21]. PCR reactions were performed in triplicate using high-fidelity Phusion^®^ DNA Polymerase (New England Biolabs, Ipswich, MA, USA). Amplicons were purified with AMPure XP beads (Beckman Coulter, Brea, CA, USA), quantified using a Qubit 3.0 fluorometer (Invitrogen, Carlsbad, CA, USA), and pooled at equimolar concentrations. All samples were sequenced in a single MiSeq run to avoid batch effects. Paired-end sequencing (2 × 300 bp) was carried out on an Illumina MiSeq platform at Gene Denovo Biotechnology Co., Ltd. (Guangzhou, China).

Raw sequences were processed using the DADA2 plugin within QIIME2 (v2020.2) [22]. Briefly, primers were removed with the cutadapt plugin, and reads were filtered, denoised, and merged to generate amplicon sequence variants (ASVs). Sequencing quality metrics were verified for all samples (Q30 > 90%), and all samples were rarefied to the same sequencing depth prior to alpha and beta diversity calculations to account for uneven sequencing depth. Taxonomy was assigned to representative ASVs using the feature-classifier plugin trained on the Greengenes database (v13_8 clustered at 99% similarity) [23]. Mitochondrial and chloroplast sequences, as well as ASVs unclassified at the kingdom level, were removed prior to downstream analysis. Alpha diversity indices (observed ASVs, Shannon, Simpson, Chao1, Faith’s phylogenetic diversity, and Pielou’s evenness) were calculated with the core-diversity plugin. Beta diversity was assessed by principal coordinates analysis (PCoA) based on Bray–Curtis, weighted/unweighted UniFrac, and Jaccard distance matrices; statistical significance among groups was tested by ANOSIM with 999 permutations. Differentially abundant taxa (from phylum to species level) were identified using the Wilcoxon rank-sum test (*p* < 0.05) after filtering out low-abundance taxa (mean relative abundance < 0.1%). Linear discriminant analysis effect size (LEfSe) was applied to identify discriminating biomarkers with an LDA score > 2.0 and *p* < 0.05 [24].

### 2.10. Correlation and Network Analysis of Multi-Omics Data

Sankey diagrams illustrating the relationships between pathways and differentially abundant metabolites (*p* < 0.05) were constructed using the ggalluvial package in R (version 4.4.2; R Foundation for Statistical Computing, Vienna, Austria) [25]. Spearman’s rank correlation analysis was performed using the cor.test function in R to evaluate associations between differentially abundant metabolites (or bacterial genera) and meat quality, growth performance, carcass traits, rumen fermentation parameters, and muscle fatty acid profiles, with stringent filtering criteria applied (|r| > 0.6 and *p* < 0.01). Pairwise correlations were assessed using Pearson’s correlation coefficients. Correlation matrices were visualized using the linkET R package, with link colors denoting correlation direction and line thickness indicating significance levels [26]. Network visualizations depicting microbe–metabolite–pathway interactions were constructed using tidygraph [27] and rendered in a concentric circle layout via ggraph [28], highlighting upregulated and downregulated nodes as well as core pathways.

### 2.11. Statistical Analysis

#### 2.11.1. Growth, Fermentation, Carcass and Serum Indices

Data were expressed as mean ± standard error of the mean (SEM). Statistical analysis was performed using the GLM procedure of SAS 9.2 (SAS Institute Inc., Cary, NC, USA). For normally distributed data, one-way ANOVA was used, followed by Duncan’s multiple range test for post hoc comparisons. Non-normally distributed data (e.g., some taxa relative abundances) were analyzed using the Kruskal–Wallis test. Significance was declared at *p* < 0.05.

#### 2.11.2. Untargeted Metabolomics Statistics

PCA and OPLS-DA were performed using the R packages gmodels and ropls, respectively, with model robustness validated by permutation testing (200 permutations) [29]. Differentially abundant metabolites between the control (CS) and each barley-replaced group (33BS, 67BS, 100BS) were selected based on variable importance in projection (VIP) > 1.0, |log_2_ fold change| ≥ 1, and *p* < 0.05 (Student’s *t*-test). Hierarchical clustering of differentially abundant metabolites was visualized as heatmaps using the pheatmap package after z-score normalization. KEGG pathway enrichment analysis was carried out on the Omicshare platform (v3.0, Gene Denovo, Guangzhou, China), with enrichment considered significant at *p* ≤ 0.05 and as a trend at 0.05 < *p* ≤ 0.10 [30]. Positive and negative ionization mode data were analyzed separately to maximize metabolite coverage.

#### 2.11.3. Rumen Microbiota Statistics

Alpha diversity indices (observed ASVs, Shannon, Simpson, Chao1, Faith’s phylogenetic diversity, and Pielou’s evenness) were calculated with the QIIME2 core-diversity plugin, and differences among groups were tested by one-way ANOVA followed by Duncan’s test. Beta diversity was assessed by PCoA based on Bray–Curtis, weighted/unweighted UniFrac, and Jaccard distance matrices; statistical significance among groups was tested by analysis of similarities (ANOSIM) with 999 permutations. Differentially abundant taxa (from phylum to species level) were identified using the Wilcoxon rank-sum test (*p* < 0.05) after filtering out low-abundance taxa (mean relative abundance < 0.1%). LEfSe was applied to identify discriminating biomarkers with an LDA score > 2.0 and *p* < 0.05 [24].

## 3. Results

### 3.1. Effect of Dietary Barley Replacement on Growth Performance

As shown in Table 2, initial body weight did not differ among the four treatment groups (*p* = 0.738). Compared with the CS group, the 33BS and 67BS groups significantly increased final BW (*p* = 0.039) and ADG (*p* = 0.041). ADFI was significantly higher in the 33BS group than in the CS group (*p* = 0.048), while the 67BS and BS groups showed intermediate values.

### 3.2. Effect of Dietary Barley Replacement on Rumen Fermentation Parameters

As shown in Table 3, compared with the CS group, the 67BS group exhibited a significantly higher ruminal ammonia nitrogen concentration (*p* = 0.019). The 33BS, 67BS, and BS groups all displayed a significant decrease in ruminal pH (*p* < 0.001). Propionate concentration was significantly increased in the 33BS and BS groups compared with the CS group (*p* = 0.001). Ruminal isobutyrate concentration tended to increase in the 67BS group (*p* = 0.085).

### 3.3. Effect of Dietary Barley Replacement on Carcass Performance

Compared with the CS group, both the 33BS and 67BS groups significantly increased carcass weight (*p* = 0.018) and carcass posterior width (*p* = 0.036) (Table 4). Loin eye area (*p* = 0.077) and carcass depth (*p* = 0.099) tended to be higher in the 33BS and 67BS groups than in the CS group.

### 3.4. Effect of Dietary Barley Replacement on Meat Quality

The BS group had a significantly lower pH in *Longissimus dorsi* compared with the CS group (*p* = 0.049) (Table 5). The 33BS, 67BS, and BS groups significantly increased a* in *Longissimus dorsi* (*p* = 0.002) and decreased L* (*p* = 0.003). No significant differences were observed in b* among groups (*p* = 0.623).

### 3.5. Effect of Dietary Barley Replacement on Nutrient Composition of Longissimus dorsi

No significant differences were observed among the dietary treatments for dry matter, crude protein, or crude fat content in either *Longissimus dorsi* (*p* > 0.05; Table 6).

### 3.6. Effect of Dietary Barley Replacement on Serum Biochemical Parameters

At day 30, the 33BS group showed significantly higher serum concentrations of TBA (*p* = 0.048), FGF-19 (*p* = 0.034), and CYP27A1 (*p* = 0.044) compared to the CS group (Table 7). The 33BS and 67BS groups exhibited a significant decrease in serum G-6-Pase concentration (*p* = 0.045).

### 3.7. Effect of Dietary Barley Replacement on Fatty Acid Composition of Longissimus dorsi

In the *Longissimus dorsi*, the 67BS group had significantly higher contents of C4:0, C6:0, C18:2t6, and C24:1 compared with the CS group (*p* < 0.05; Table 8), whereas C18:0 content was significantly lower in the 67BS group than in the CS group (*p* < 0.05). The 33BS group exhibited the highest C18:3n3 content among all groups (*p* < 0.01). Both the 67BS and BS groups showed significantly greater C22:6n3 levels relative to the CS group (*p* < 0.05). No significant differences were detected among treatments for total SFA, UFA, MUFA, PUFA, n-3 PUFA, n-6 PUFA, or the n-6/n-3 and U/S ratios (*p* > 0.05).

### 3.8. Multivariate Analysis of the Muscle Metabolome

#### 3.8.1. Comparative Sample Analysis

**Figure 1:**
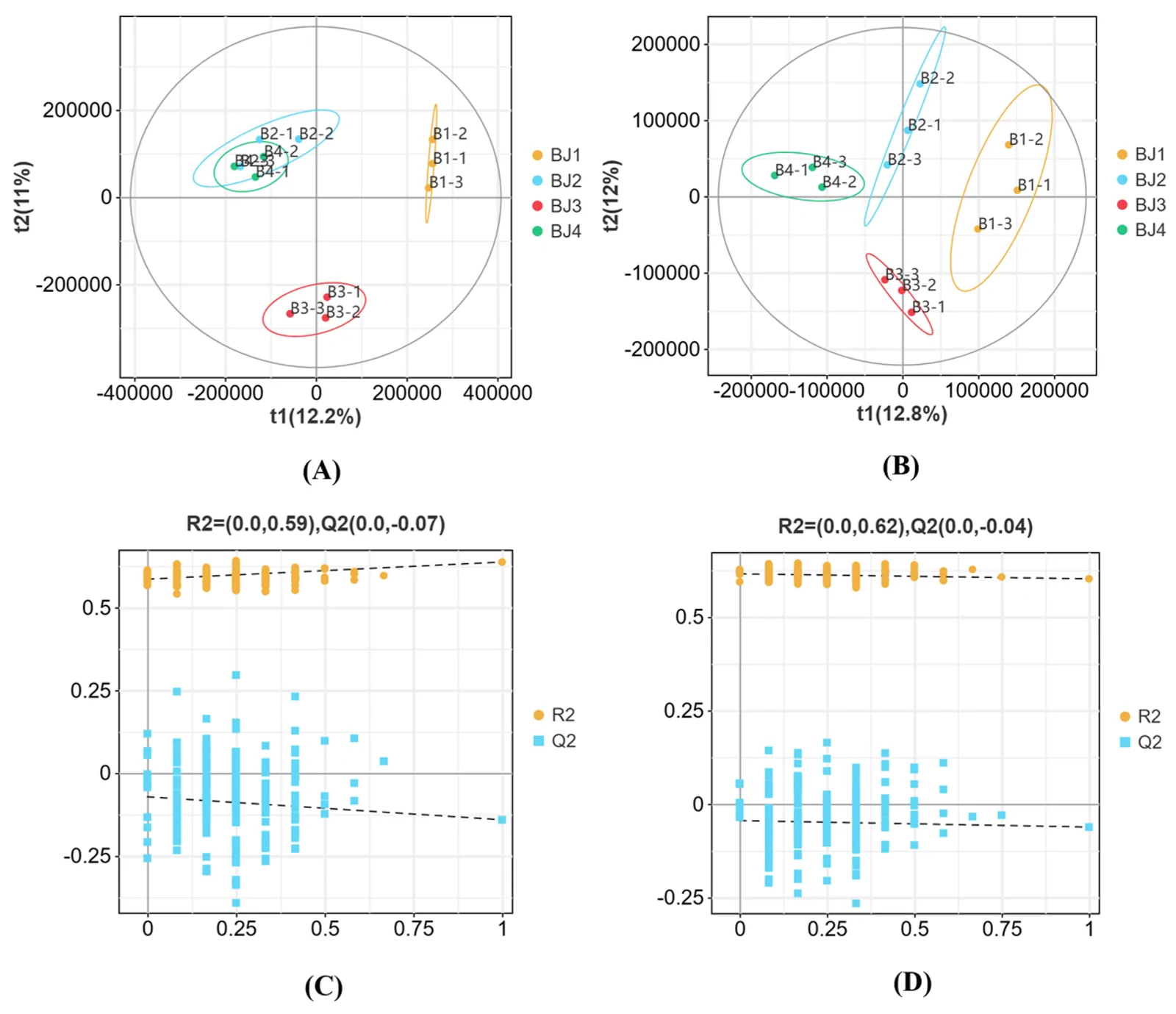
Partial least squares discriminant analysis (PLS-DA) score plots and permutation test validation of *Longissimus dorsi* from cashmere goats fed four graded barley replacement levels. (**A**,**C**) Positive ion mode (POS); (**B**,**D**) negative ion mode (NEG). BJ1: corn–soybean meal basal diet (0% barley); BJ2: 33% barley starch replacement; BJ3: 67% barley starch replacement; BJ4: 100% barley starch replacement.

PLS-DA score plots revealed that under both positive (Figure 1A) and negative (Figure 1B) ion modes, all 12 samples from the four treatment groups fell within the 95% Hotelling’s T^2^ ellipse, with QC samples tightly clustered, confirming the stability of the LC-MS platform and the reliability of the acquired data. A clear separation trend was observed among the dietary treatment groups, indicating that the dietary treatments exerted distinct effects on the metabolomic profiles of *Longissimus dorsi*, with the B4 group deviating most markedly from the B1 group.

Permutation testing (200 random permutations) confirmed that all PLS-DA models were robust and free from overfitting. Under positive ion mode (POS) (Figure 1C), the intercepts of R^2^ and Q^2^ were 0.0 and −0.07, respectively (R^2^ = 0.59, Q^2^ = −0.07). Under negative ion mode (NEG) (Figure 1D), the corresponding intercepts were 0.0 and −0.04 (R^2^ = 0.62, Q^2^ = −0.04). The negative Q^2^ intercept values in both ionization modes, coupled with all permuted Q^2^ values falling below the original model Q^2^ values, collectively demonstrated that the observed group separations reflected genuine biological differences rather than random classification, thereby validating the reliability of the supervised models.

#### 3.8.2. Differential Metabolite Screening in *Longissimus dorsi*

**Figure 2:**
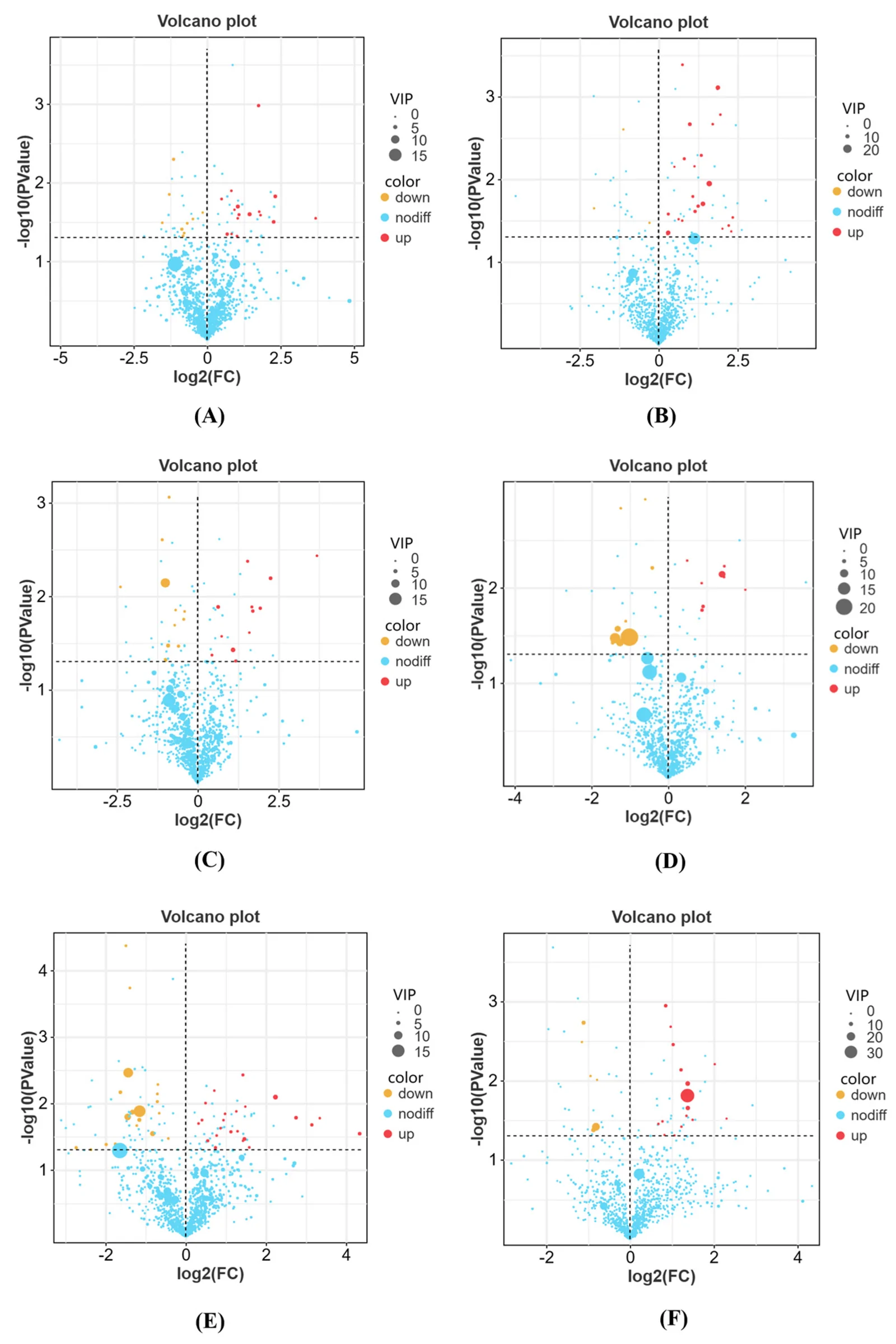
Statistics of differentially abundant metabolites in the *Longissimus dorsi* of cashmere goats fed diets with different barley replacement levels. (**A**,**C**,**E**) Volcano plots showing the numbers of upregulated and downregulated differentially abundant metabolites identified under negative (NEG) ion mode in the B2 vs. B1, B3 vs. B1, and B4 vs. B1 comparisons, respectively. (**B**,**D**,**F**) Volcano plots showing the numbers of upregulated and downregulated differentially abundant metabolites identified under positive (POS) ion mode in the B2 vs. B1, B3 vs. B1, and B4 vs. B1 comparisons, respectively. Differentially abundant metabolites were screened based on VIP > 1.0, |log_2_ fold change| ≥ 1, and *p* < 0.05.

Under NEG ion mode, compared with the B1 group, the B2 group exhibited 17 upregulated and 9 downregulated differential metabolites, the B3 group showed 12 upregulated and 12 downregulated, and the B4 group displayed 24 upregulated and 20 downregulated (Figure 2A,C,E). Under POS ion mode, relative to the B1 group, 24 upregulated and 3 downregulated differential metabolites were identified in the B2 group, 8 upregulated and 10 downregulated in the B3 group, and 16 upregulated and 6 downregulated in the B4 group (Figure 2B,D,F).

#### 3.8.3. KEGG Pathway Enrichment Analysis of Differential Metabolites

**Figure 3:**
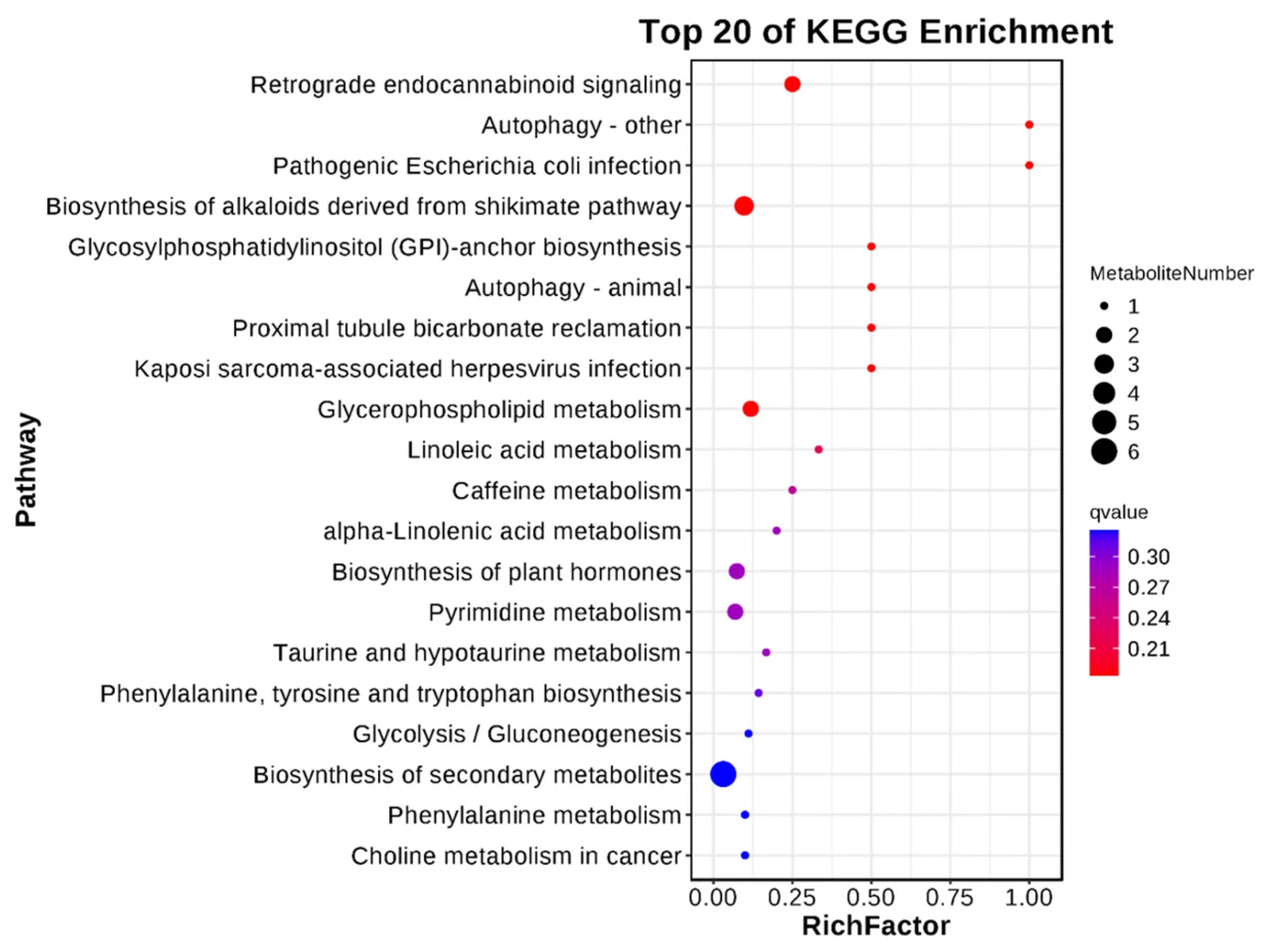
Top 20 enriched KEGG pathways of differentially abundant metabolites in *Longissimus dorsi* between the B1 and B2 groups of cashmere goats.

KEGG pathway enrichment analysis showed that, relative to the B1 group, a total of 36 pathways were enriched from the differential metabolites in the Longissimus dorsi of the B2 group. The top 20 most significantly enriched pathways are displayed in Figure 3, including retrograde endocannabinoid signaling (*p* = 0.0010), pathogenic Escherichia coli infection (*p* = 0.0029), biosynthesis of alkaloids derived from the shikimate pathway (*p* = 0.0226), glycosylphosphatidylinositol (GPI) anchor biosynthesis (*p* = 0.0414), proximal tubule bicarbonate reclamation (*p* = 0.0414), glycerophospholipid metabolism (*p* = 0.0459), linoleic acid metabolism (*p* = 0.0616), caffeine metabolism (*p* = 0.0813), and α-linolenic acid metabolism (*p* = 0.1007). Detailed information on the KEGG enrichment analysis is provided in Table 9.

#### 3.8.4. Comparative KEGG Pathway Enrichment Across Barley Substitution Levels

**Figure 4:**
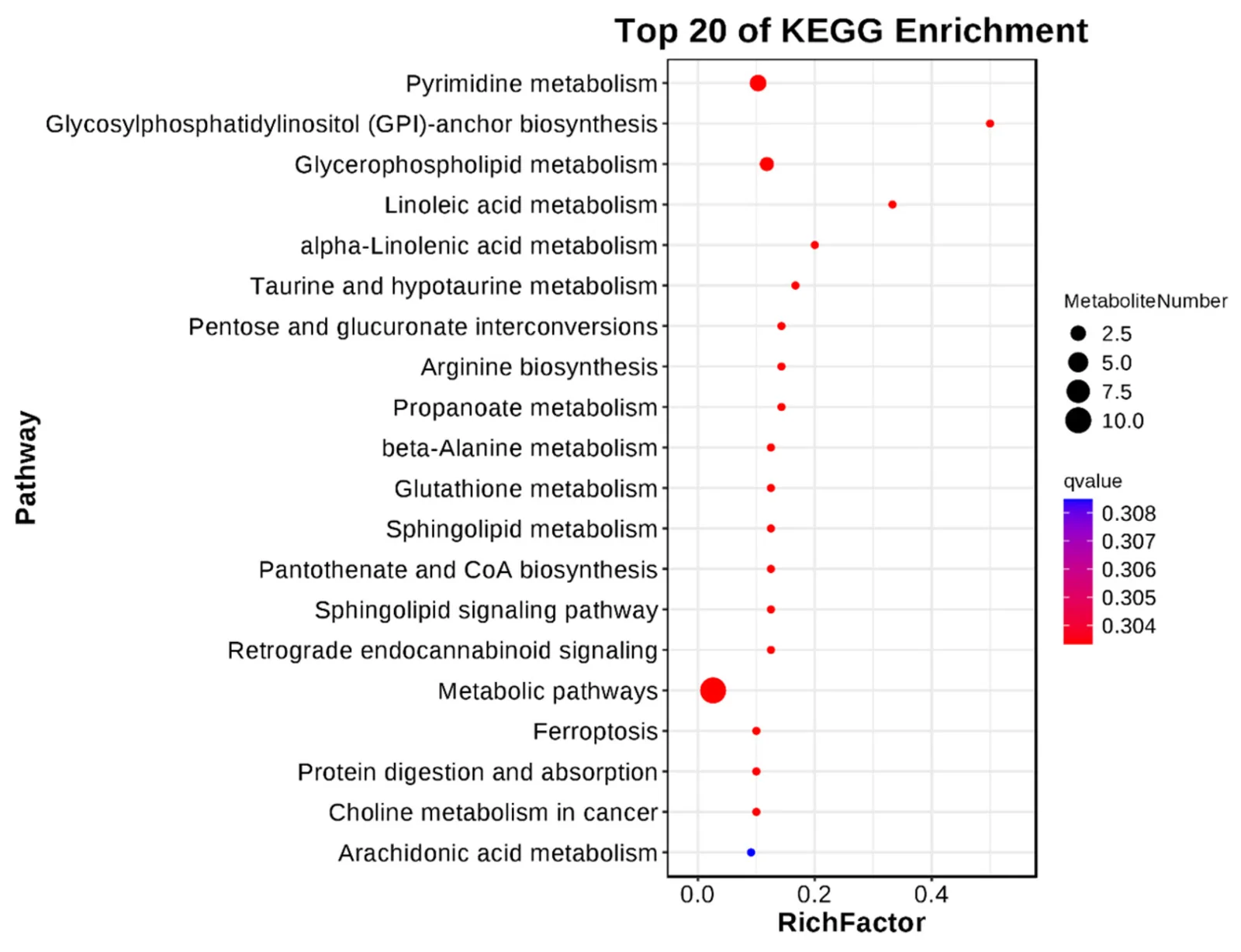
Top 20 enriched KEGG pathways of differentially abundant metabolites in *Longissimus dorsi* between the B1 and B3 groups of cashmere goats.

**Figure 5:**
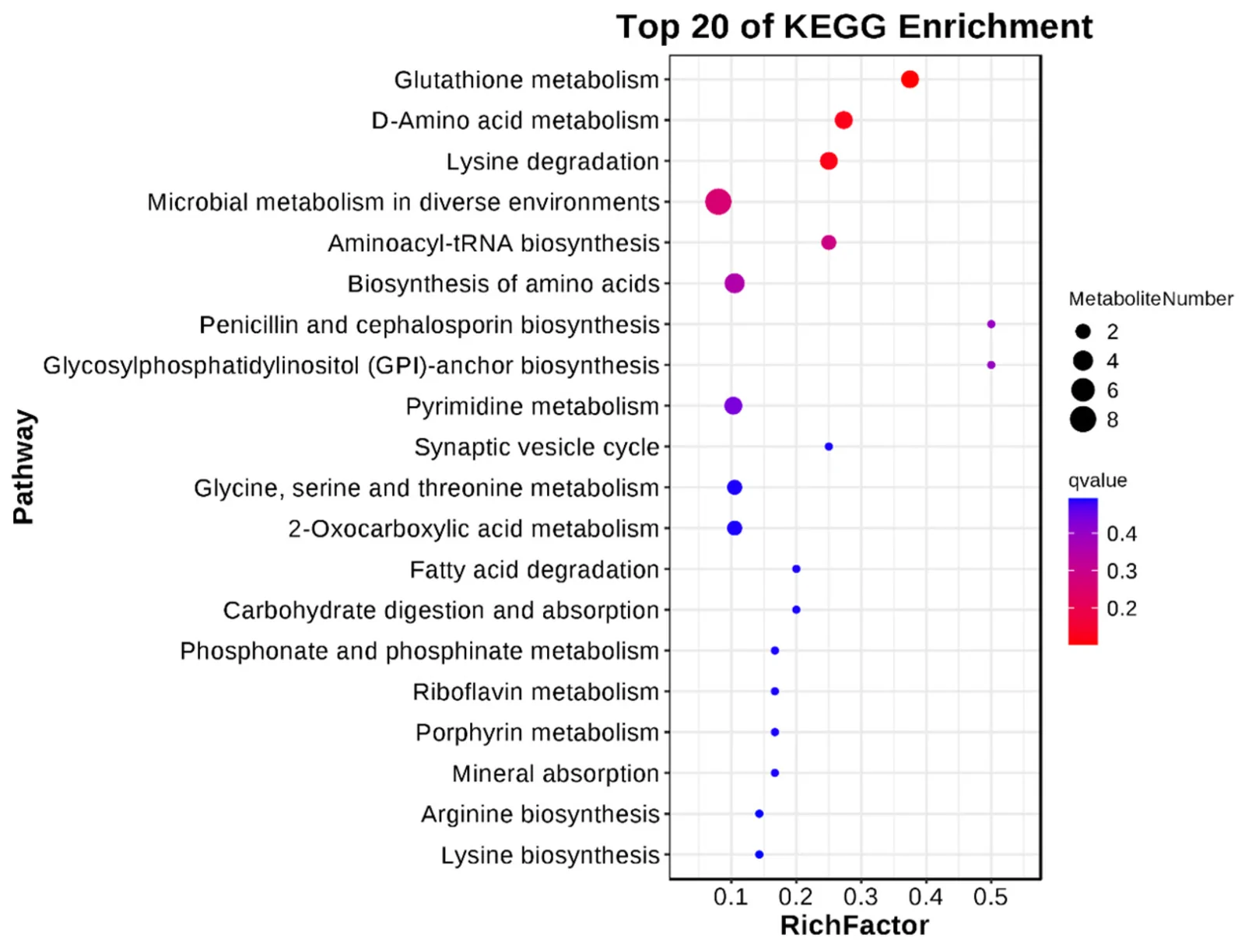
Top 20 enriched KEGG pathways of differentially abundant metabolites in *Longissimus dorsi* between the B1 and B4 groups of cashmere goats.

As shown in Figure 3, Figure 4 and Figure 5, differential metabolites in the *Longissimus dorsi* were compared across three dietary barley-for-corn substitution levels (B2, B3, B4 vs. B1 control). In the B2 vs. B1 comparison, the most notable feature was the upregulation of two glycerophospholipids—PE(P-18:1/20:4) and PC(16:0/16:0)—which appeared jointly in retrograde endocannabinoid signaling, autophagy, and glycerophospholipid metabolism pathways (Table 9). Emetine, shikimate and 3-methylxanthine were also upregulated, while alpha-D-glucose was the sole downregulated metabolite. In the B3 vs. B1 comparison, PC(16:0/16:0) remained elevated, and beta-alanine was upregulated in retrograde endocannabinoid signaling, glycerophospholipid metabolism and linoleic acid metabolism (Table 10). Guanidinoethyl sulfonate and digalacturonic acid were also increased. Among the downregulated metabolites, O-phosphoethanolamine and the nucleotide monophosphates 2′-deoxycytidine 5′-monophosphate and thymidine decreased, and gamma-glutamylcysteine appeared as a newly downregulated metabolite. The B4 vs. B1 comparison exhibited the broadest metabolic changes, with glutathione metabolism, D-amino acid metabolism, lysine degradation and biosynthesis of amino acids as the dominant enriched pathways (Table 11). Glycine was the most extensively downregulated metabolite, being reduced in seven pathways, including glutathione metabolism, D-amino acid metabolism, lysine degradation, aminoacyl-tRNA biosynthesis, glycine–serine–threonine metabolism and the synaptic vesicle cycle. Gamma-glutamylcysteine and L-pyroglutamic acid were concurrently dowregulated in glutathione metabolism. Upregulated metabolites in B4 comprised phosphoserine, glutaric acid, cis-4-hydroxy-D-proline, deoxythymidine triphosphate, uridine 5′-monophosphate, 4-chlorophenoxyacetic acid and gallic acid.

### 3.9. Rumen 16S rRNA Gene Amplicon Sequencing and Analysis

#### 3.9.1. Effects of Different Barley Replacement Levels on Rumen Microbial Community Diversity in Cashmere Goats

**Figure 6:**
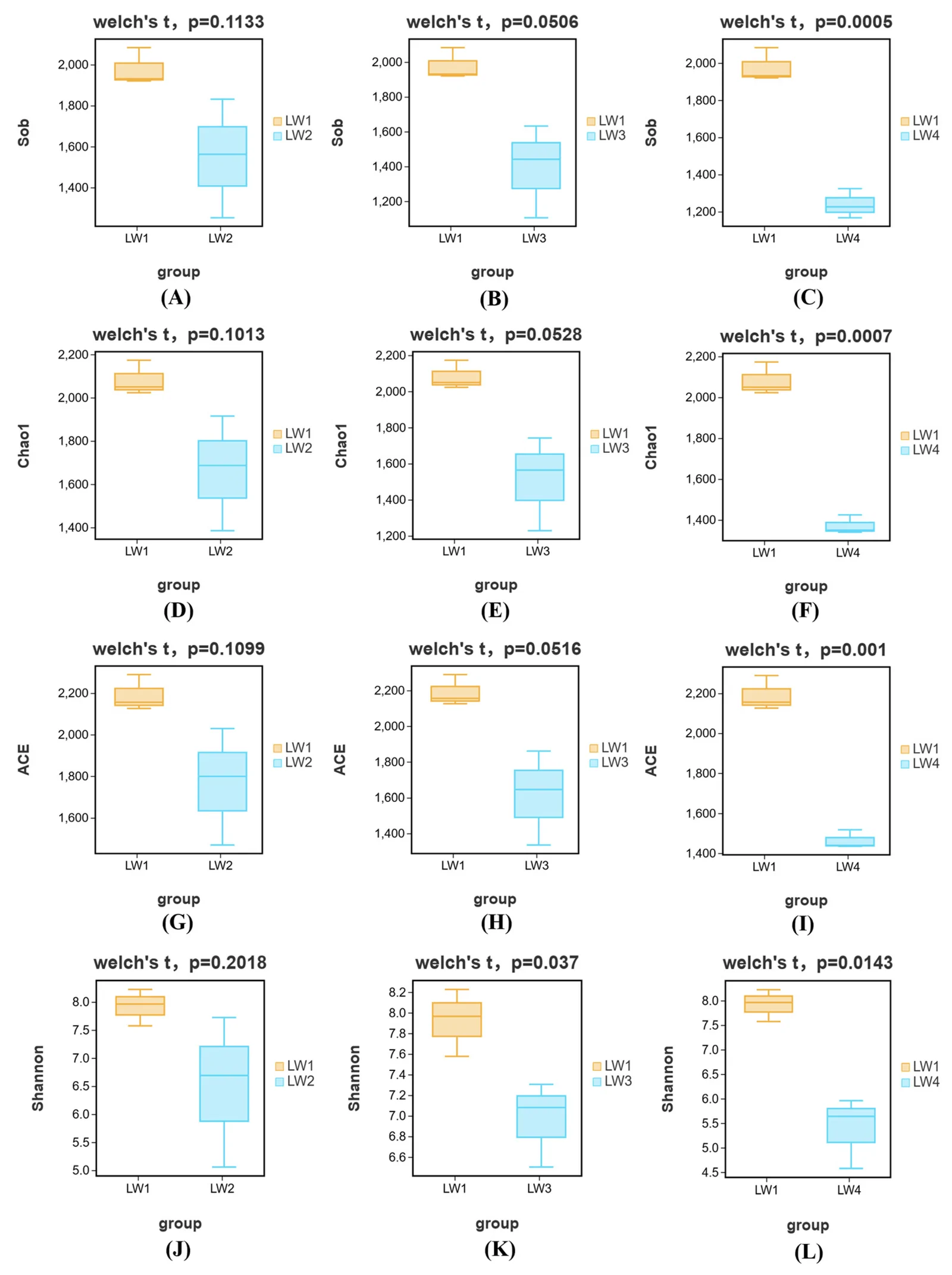
Comparison of rumen microbial alpha diversity indices between the experimental groups and the control group. (**A**–**C**) Box plots of α-diversity Sobs (observed species) index with Welch’s *t*-test for B2 vs. B1, B3 vs. B1, and B4 vs. B1, respectively. (**D**–**F**) Box plots of α-diversity Chao1 index with Welch’s *t*-test for B2 vs. B1, B3 vs. B1, and B4 vs. B1, respectively. (**G**–**I**) Box plots of α-diversity ACE index with Welch’s *t*-test for B2 vs. B1, B3 vs. B1, and B4 vs. B1, respectively. (**J**–**L**) Box plots of α-diversity Shannon index with Welch’s *t*-test for B2 vs. B1, B3 vs. B1, and B4 vs. B1, respectively.

The results of the intestinal microbial alpha diversity analysis are presented in Figure 6. Compared with the control group (LW1), no significant differences were observed in the Sobs, Chao1, ACE, and Shannon indices in the LW2 treatment group (*p* > 0.05). In the LW3 group, the Shannon index was significantly decreased (*p* = 0.037), while the Sobs, Chao1, and ACE indices exhibited decreasing trends (0.05 < *p* < 0.10). In the LW4 group, all alpha diversity indices were significantly lower than those in the control group (*p* < 0.01), with *p* = 0.0005 for Sobs, *p* = 0.0007 for Chao1, *p* = 0.001 for ACE, and *p* = 0.0143 for the Shannon index. These results indicate that the LW3 and LW4 treatments significantly reduced the diversity of the intestinal microbial community, with a more pronounced suppressive effect observed in the LW4 group.

**Figure 7:**
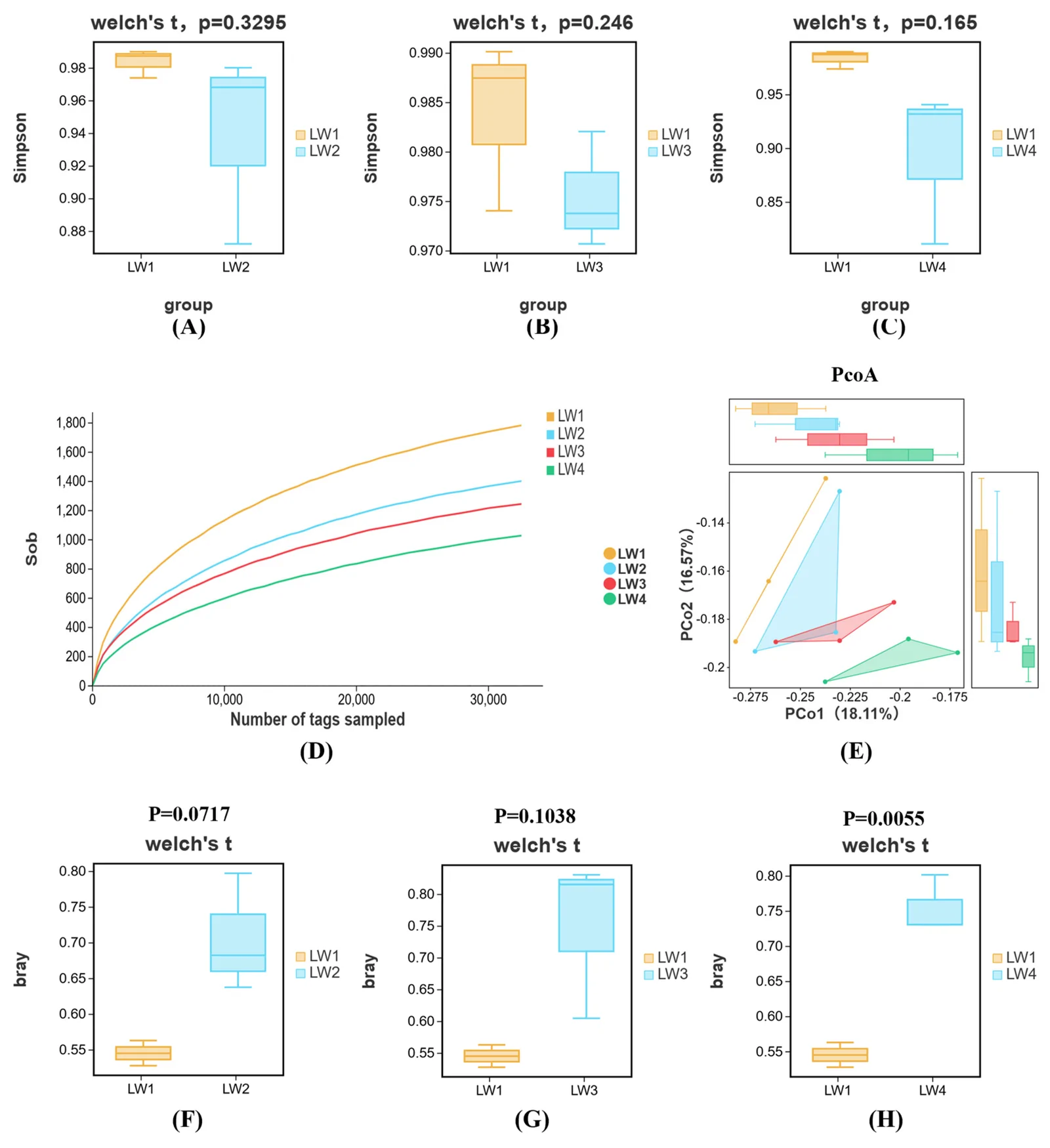
Multivariate statistical analysis comparing the differences in rumen microbial alpha diversity indices and beta diversity between the experimental groups and the control group. (**A**–**C**) Box plots of α-diversity Simpson index with Welch’s *t*-test for B2 vs. B1, B3 vs. B1, and B4 vs. B1, respectively. (**D**) Sobs rarefaction curves for the four groups. (**E**) Principal coordinates analysis (PCoA) plot of β-diversity for the four groups. (**F**–**H**) Box plots of β-diversity (Bray–Curtis dissimilarity) with Welch’s *t*-test for B2 vs. B1, B3 vs. B1, and B4 vs. B1, respectively.

The results of the rumen microbial community evenness and overall structure analysis are presented in Figure 7. The Simpson index showed no significant differences in community evenness between any treatment group and the control group (LW1) (*p* > 0.05), indicating that the treatments did not significantly alter the abundance distribution of dominant species. The rarefaction curves approached a plateau, suggesting that the sequencing depth in this study covered the vast majority of species and that data saturation was adequate. Principal coordinates analysis (PCoA) based on Bray–Curtis distance revealed a clear separation of the LW4 group from the control group, while the distribution of the LW2 and LW3 groups partially overlapped with that of the control. Further intergroup difference testing showed that the Bray–Curtis distance between the LW4 group and the control group was highly significant (*p* = 0.0055), the LW2 group exhibited a tendency toward significance (*p* = 0.0717), and no statistically significant difference was observed for the LW3 group (*p* = 0.1038). These results indicate that the LW4 treatment significantly altered the overall community structure of the host rumen microbiota, whereas the LW2 and LW3 treatments had relatively weak effects on community structure.

#### 3.9.2. Species Composition Analysis and Functional Characterization

**Figure 8:**
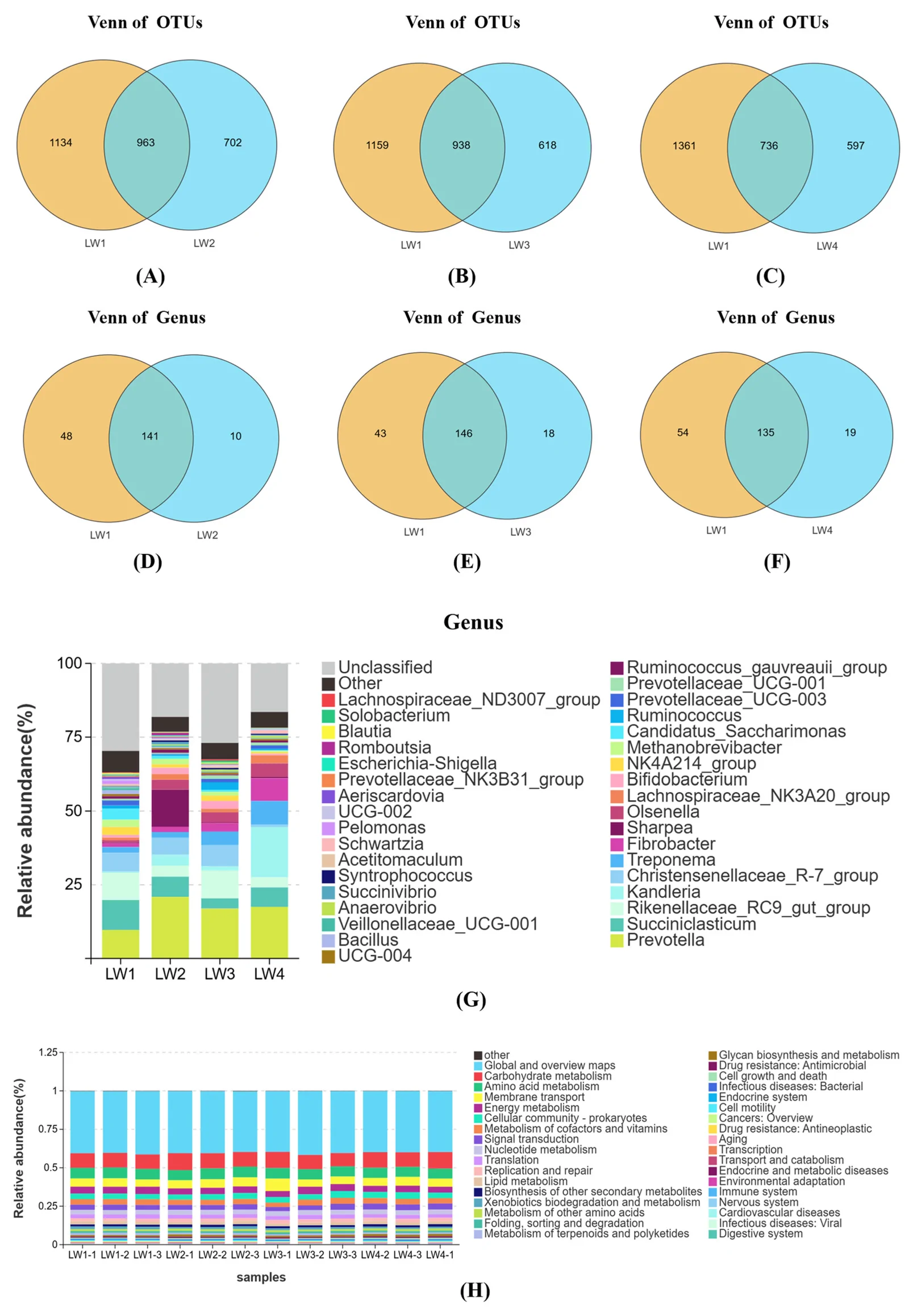
Venn analysis of differential taxa, taxonomic composition, and functional profiling among the four groups. (**A**–**C**) Venn diagrams of shared and unique OTUs between B2 and B1, B3 and B1, and B4 and B1, respectively. (**D**–**F**) Venn diagrams of shared and unique genera between B2 and B1, B3 and B1, and B4 and B1, respectively. (**G**) Stacked bar plot of genus-level taxonomic composition for the four groups. (**H**) Stacked bar plot of Tax4Fun-predicted functional profiles for the four groups.

The results of the rumen microbial species composition and functional prediction are presented in Figure 8. The OTU-level Venn diagrams revealed that the number of shared OTUs between the LW4 group and the control group was markedly reduced, accompanied by a decrease in unique OTUs, whereas the LW2 and LW3 groups exhibited high species overlap with the control. The genus-level Venn diagrams further corroborated this trend, showing a substantially lower number of shared genera in the LW4 group compared with the other groups. Genus-level community composition analysis indicated that *Prevotella*, *Bacteroides*, and *Rikenellaceae_RC9_gut_group* were the core dominant genera across all groups. Notably, the relative abundance of *Prevotella* was significantly increased in the LW2 group and significantly decreased in the LW4 group. KEGG functional prediction revealed that the distribution of core metabolic pathways (carbohydrate metabolism, amino acid metabolism, and energy metabolism) was highly similar among groups, with no significant differences in functional pathways, suggesting a certain degree of functional redundancy in the microbiota. These results demonstrate that the LW4 treatment significantly altered the species composition of the rumen microbial community without markedly affecting core nutrient metabolic functions, whereas the LW2 treatment promoted the enrichment of the fiber-degrading bacterium *Prevotella*, potentially exerting a positive effect on fiber digestion and utilization in the host. 

#### 3.9.3. Differential Abundance Analysis of Bacterial Communities Among Groups

**Figure 9:**
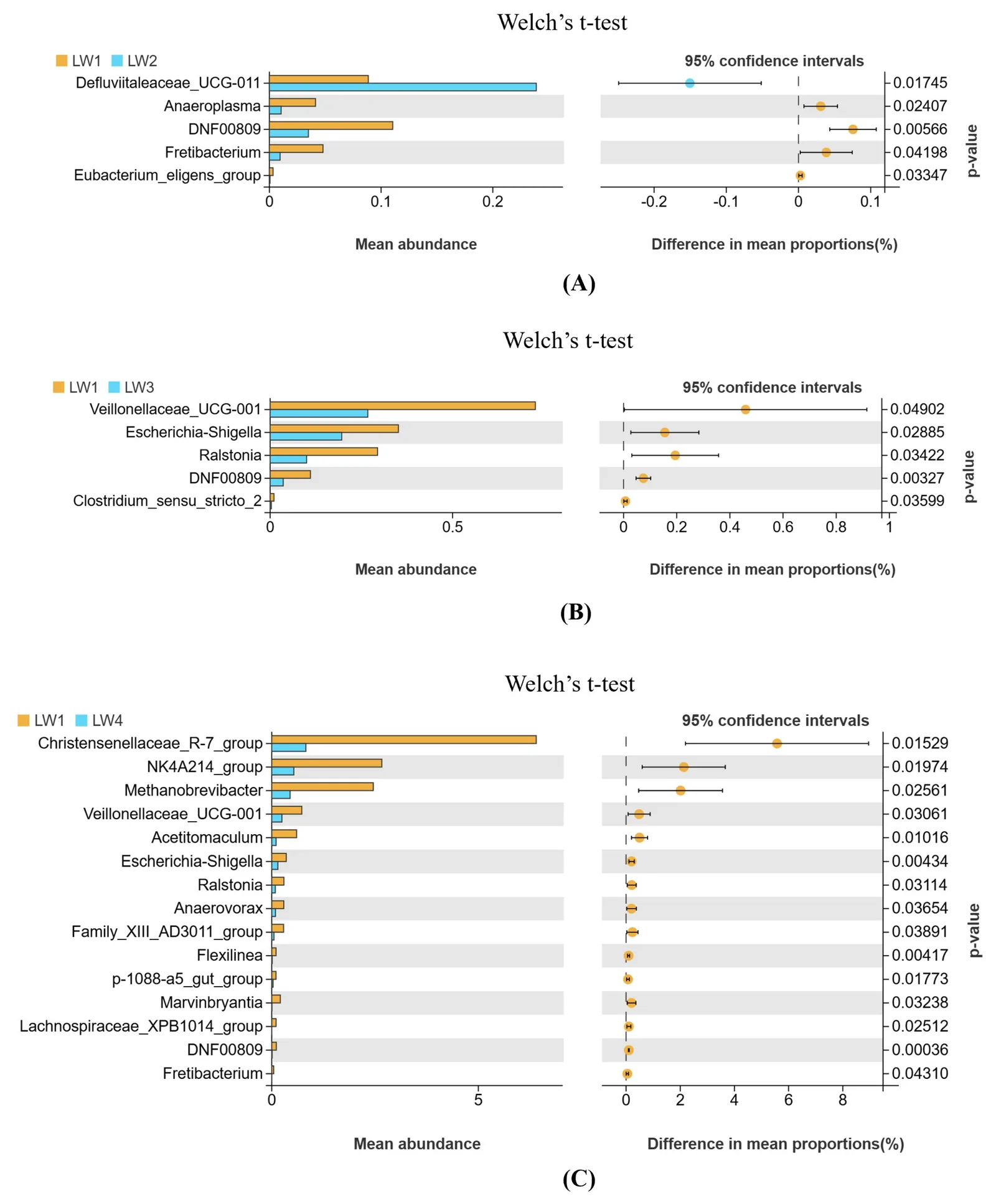
Differential abundance analysis of bacterial genera among the four groups. (**A**) Bar chart of Welch’s *t*-test for differential relative abundance of bacterial genera between B2 and B1. (**B**) Bar chart of Welch’s *t*-test for differential relative abundance of bacterial genera between B3 and B1. (**C**) Bar chart of Welch’s *t*-test for differential relative abundance of bacterial genera between B4 and B1.

As shown in Figure 9, Welch’s *t*-test was used to identify genera with significant differences in relative abundance between the control group (LW1) and each treatment group (LW2, LW3, and LW4) (*p* < 0.05), and the results are shown in Figure 4. Compared with LW1, a total of 5 genera in LW2, 5 in LW3, and 13 in LW4 showed significant changes, indicating that the treatments exerted varying degrees of perturbation on the rumen bacterial community structure. 

In the LW2 group, the relative abundance of *Defluviitaleaceae_UCG-011* was significantly increased (*p* = 0.017), whereas those of *Anaeroplasma*, *DNF00809*, *Fretibacterium*, and *Eubacterium eligens* group were significantly decreased (*p* < 0.05). All five differentially abundant genera identified in the LW3 group exhibited decreased relative abundances, including *Veillonellaceae_UCG-001*, *Escherichia-Shigella*, *Ralstonia*, *DNF00809*, and *Clostridium sensu stricto 2* (*p* < 0.05). In the LW4 group, all significantly changed genera showed lower relative abundances in the treatment group than in the control group. Genera with substantial decreases included *Christensenellaceae_R-7_group* (6.40% vs. 0.83%, *p* = 0.015), *NK4A214_group* (2.66% vs. 0.53%, *p* = 0.020), *Methanobrevibacter* (2.46% vs. 0.44%, *p* = 0.026), as well as *Veillonellaceae_UCG-001*, *Acetitomaculum*, *Escherichia-Shigella*, *Ralstonia*, *Anaerovorax*, *Flexilinea*, and *Marvinbryantia* (all *p* < 0.05). Notably, *DNF00809* was consistently and significantly decreased across all three treatment groups, whereas *Veillonellaceae_UCG-001*, *Escherichia-Shigella*, and *Ralstonia* were commonly decreased in both the LW3 and LW4 groups. These results indicate that the experimental treatments significantly reshaped the rumen bacterial community, with the most extensive changes observed in the LW4 group.

#### 3.9.4. Correlation Analysis Between Key Indicators and Host Phenotypic Traits

**Figure 10:**
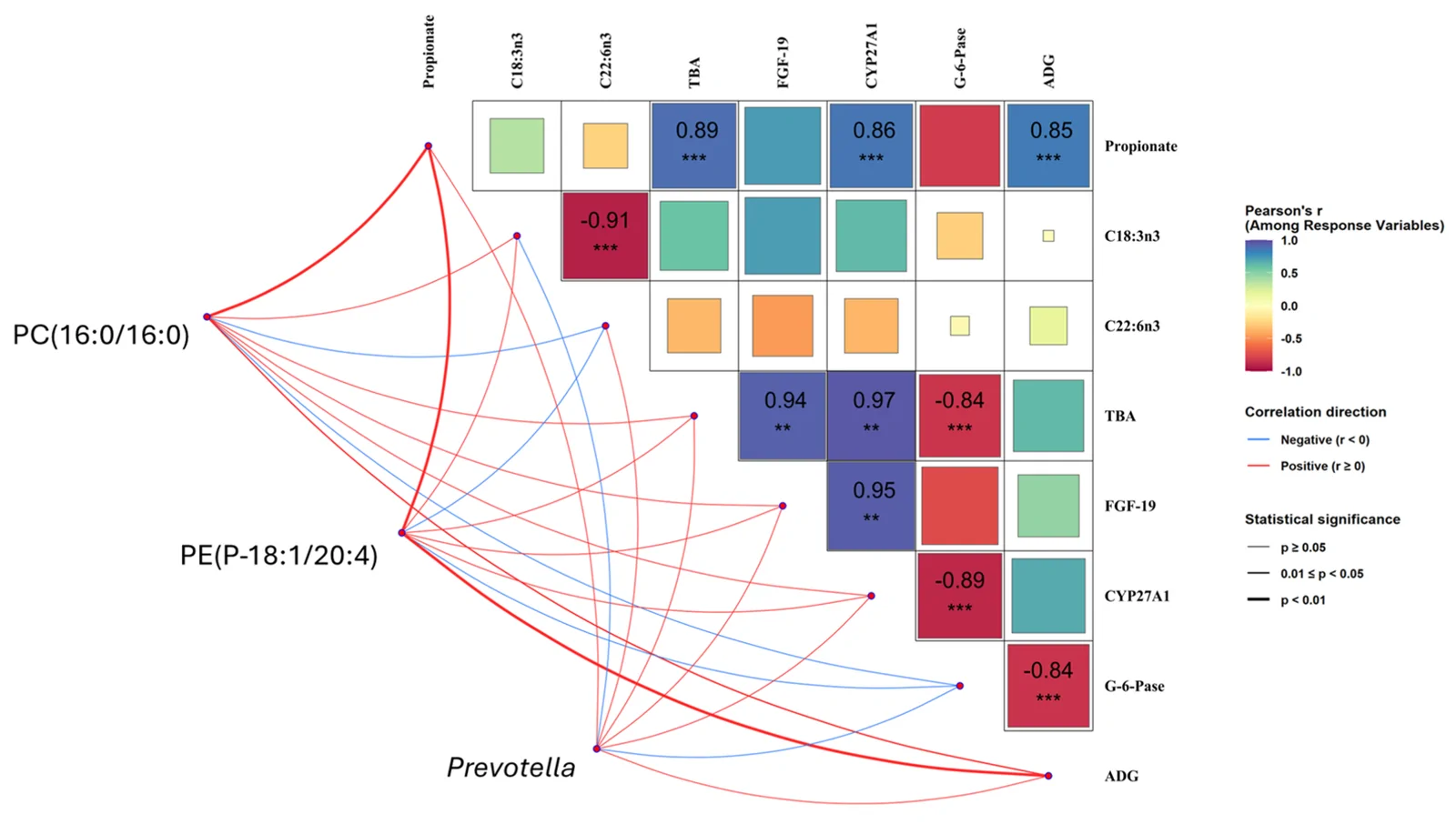
Correlation heatmap showing associations of key indicators (PC(16:0/16:0), PE(P-18:1/20:4), and Prevotella) with growth performance, fatty acid profile, and serum biochemical parameters in cashmere goats. The heatmap displays Pearson correlation coefficients among eight response variables (propionate, C18:3n3, C22:6n3, TBA, FGF-19, CYP27A1, G-6-Pase, and ADG). Red and blue colors indicate positive and negative correlations, respectively, with a gradient transition. Network lines represent Spearman’s rank correlations between the three indicators and each response variable. Line color denotes the direction of association (red, positive; blue, negative), while line thickness reflects statistical significance. Significance levels are indicated as follows: ** *p* < 0.01, *** *p* < 0.001.

As shown in Figure 10, Spearman’s rank correlation analysis revealed potential associations between key indicators and host phenotypic traits. Both PE(P-18:1/20:4) and PC(16:0/16:0) were significantly positively correlated with ruminal propionate concentration (r = 0.943, *p* = 0.005) and average daily gain (ADG; r = 0.986, *p* < 0.001 and r = 0.841, *p* = 0.036, respectively). These statistical associations indicate that muscle glycerophospholipid levels vary in parallel with ruminal propionate and growth performance, but correlation alone does not establish a mechanistic or causal linkage. Furthermore, PE(P-18:1/20:4) and PC(16:0/16:0) showed strong positive correlation trends with serum CYP27A1, FGF-19, and total bile acid (TBA) levels (r = 0.657–0.771), and negative correlation trends with glucose-6-phosphatase (G-6-Pase; r = −0.657 to −0.714). Although these trends did not reach statistical significance, the overall pattern is compatible with a coordinated relationship between glycerophospholipid profiles and circulating markers of bile acid metabolism and gluconeogenesis. Notably, the relative abundance of Prevotella exhibited a strong negative trend with G-6-Pase (r = −0.771, *p* = 0.072) and positive trends with CYP27A1, FGF-19, and TBA (r = 0.314–0.371), which were likewise not statistically significant. The correlations of Prevotella with propionate and ADG were relatively weak (r = 0.371–0.435), and none of the three indicators showed significant correlations with muscular C18:3n-3 or C22:6n-3 contents, implying that under the 33% barley replacement condition, the inhibition of biohydrogenation may not yet be fully manifested, or that fatty acid deposition is subject to multifactorial regulation. Collectively, these correlation analyses reveal consistent pairwise associations among muscle glycerophospholipid levels, *Prevotella* relative abundance, and host energy-related phenotypes in the 33BS group. Given the correlative nature of the analysis and the limited sample size for omics data, no causal or mechanistic inference can be drawn; the results are presented solely as descriptive associations for hypothesis generation.

## 4. Discussion

The present study systematically evaluated the effects of graded dietary replacement of corn starch with barley starch on growth performance, rumen fermentation, carcass traits, meat quality, serum bile acid-related parameters, muscle fatty acid profiles, and the rumen microbiome in Arbas White Cashmere goats. Furthermore, untargeted muscle metabolomics was applied to elucidate the metabolic signatures underlying meat quality alterations. Our findings demonstrate that partial barley substitution, particularly at 33% and 67% levels, improved growth performance and carcass traits without compromising rumen health. Conversely, total replacement (100% barley starch) depressed ruminal microbial diversity and markedly altered the bacterial community, accompanied by distinct shifts in muscle metabolism. These results support our hypothesis and identify a moderate barley inclusion level as the optimal feeding strategy.

### 4.1. Effects of Barley Replacement on Growth Performance and Rumen Fermentation

In the current study, goats fed the 33BS and 67BS diets exhibited greater final BW and ADG compared with those fed the corn-based control diet, with the 33BS group also demonstrating a significant increase in ADFI. This growth-promoting effect at moderate barley inclusion is consistent with previous reports in lambs and beef cattle, where barley improved palatability and energy availability due to its more rapidly fermentable starch fraction [31,32]. Because barley starch is fermented faster than corn starch in the rumen, it can shift VFA profiles toward propionate, which would be expected to increase gluconeogenic energy supply to the host [33]. Indeed, the 33BS and 67BS groups exhibited significantly higher propionate concentrations. As propionate is a primary gluconeogenic precursor in ruminants, the higher propionate levels observed align with the improved ADG in these groups, although other contributing factors cannot be excluded.

All barley-containing diets decreased ruminal pH, with the most pronounced decline observed in the 67BS group. However, mean pH values remained above 5.0, suggesting that the risk of subacute ruminal acidosis was limited under the current feeding regimen [34]. In contrast, the elevated ruminal ammonia nitrogen concentration in the 67BS group indicates a higher rate of protein degradation or reduced efficiency of microbial protein synthesis. This potentially reflects an uncoupling between the availability of readily fermentable carbohydrates and dietary protein. These findings suggest that while moderate barley inclusion optimizes rumen fermentation, excessive substitution may disrupt nitrogen utilization efficiency.

### 4.2. Rumen Microbial Community Responses

The observed shifts in rumen fermentation were closely associated with changes in the ruminal microbiota. Alpha diversity analysis revealed that the complete substitution (BS) group exhibited a significant reduction in the Sobs, Chao1, ACE, and Shannon indices, whereas the 33BS and 67BS groups showed only marginal or moderate changes. Beta diversity analysis based on Bray–Curtis distances further demonstrated a clear separation of the BS group from the control, while the 33BS and 67BS groups overlapped partially with the control. This highlights that only the all-barley diet profoundly restructured the rumen microbiota, consistent with the view that excessive inclusion of rapidly fermentable starch can disrupt the ecological balance of the ruminal microbial community [35].

At the genus level, Prevotella, Bacteroides, and the *Rikenellaceae_RC9_gut_group* were identified as the core dominant taxa across all groups, aligning with their well-established roles in polysaccharide degradation [36]. Notably, the relative abundance of *Prevotella* was significantly enriched in the 33BS group but sharply decreased in the BS group. Prevotella species are highly capable of degrading starch and hemicellulose to produce propionate [37]; the observed enrichment of *Prevotella* in the 33BS group is therefore consistent with the higher ruminal propionate concentrations measured in this treatment, although a causal role cannot be confirmed from the present correlative data.

Conversely, the BS group experienced a broad depletion of multiple fibrolytic and hydrogenotrophic genera, including the *Christensenellaceae_R-7_group*, *NK4A214_group*, *Methanobrevibacter*, and *Veillonellaceae_UCG-001*. Many of these taxa are essential for fiber degradation and interspecies hydrogen transfer; their decline likely reflects ecological disruption driven by the rapid fermentation of the all-barley diet [38]. Despite these taxonomic shifts, KEGG functional predictions showed no significant differences in core metabolic pathways among the groups, suggesting functional redundancy that maintained the overall digestive capacity of the rumen microbiota [39]. Furthermore, Welch’s *t*-test identified *DNF00809* as a consistently depleted genus across all three barley-replaced groups, whereas *Veillonellaceae_UCG-001*, *Escherichia-Shigella*, and *Ralstonia* were commonly decreased in both the 67BS and BS groups. The broadest taxonomic perturbation was observed in the BS group, with 13 significantly altered genera, firmly supporting the notion that excessive barley substitution destabilizes the rumen ecosystem.

### 4.3. Carcass Traits and Meat Quality

Carcass weight and posterior width were significantly greater in the 33BS and 67BS groups than in the control, and loin eye area tended to increase, indicating an overall improvement in muscling. These enhancements are compatible with the more efficient energy harvest expected from barley-driven propionate fermentation [40]. The BS group displayed a lower ultimate pH in the *Longissimus dorsi*, which may be associated with increased glycogenolysis in the early postmortem period, possibly due to altered ante-mortem energy metabolism [41]. Interestingly, a* consistently increased, while L* decreased in both the *Longissimus dorsi* and biceps femoris across all barley-fed groups, resulting in a darker and redder meat color. The specific mechanisms underlying these color changes warrant further investigation but may be related to altered myoglobin concentrations or shifts in muscle oxidative status [42].

### 4.4. Muscle Fatty Acid Composition

Dietary barley inclusion altered the intramuscular fatty acid profile, although the total proportions of SFA, MUFA, and PUFA were not significantly modified. The 67BS treatment increased the contents of short-chain fatty acids (C4:0 and C6:0) as well as the long-chain n-3 PUFA C22:6n-3 (DHA). The accumulation of short-chain fatty acids in muscle is atypical and might reflect the ruminal production of butyrate and caproate, which can be incorporated into tissue lipids post-absorption [43]. The increase in DHA is of particular nutritional interest, as n-3 fatty acids offer potential health benefits for consumers [44]. Additionally, the 33BS group exhibited the highest proportion of C18:3n-3 (alpha-linolenic acid), an essential fatty acid. The biological basis for this increase remains to be established, and several non-exclusive explanations can be proposed. First, intrinsic differences in fatty acid composition between barley and corn grain may directly contribute to the altered muscle C18:3n-3 content. Second, moderate barley inclusion may modify ruminal passage rate and digesta retention time, increasing the fraction of dietary C18:3n-3 that escapes complete ruminal biohydrogenation. Third, shifts in ruminal microbial composition, including the enrichment of *Prevotella*, could alter biohydrogenation pathways and metabolic fluxes, though this remains speculative and requires direct validation [45]. Meanwhile, the BS group also showed significantly elevated DHA, which may be associated with a disrupted rumen environment and altered biohydrogenation activity, though this interpretation also remains correlative.

### 4.5. Muscle Metabolomics Reveals Metabolic Reprogramming

Untargeted metabolomic analysis provided deeper insights into the metabolic consequences of barley replacement. PLS-DA clearly separated the BS group from the control, whereas the 33BS and 67BS groups displayed milder metabolic divergence. This indicates that the extent of metabolic reprogramming in the muscle was proportional to the dietary barley inclusion level. Pathway enrichment analysis revealed that moderate substitution (33BS and 67BS) predominantly affected glycerophospholipid metabolism and retrograde endocannabinoid signaling, characterized by the upregulation of metabolites such as PE(P-18:1_20:4) and PC(16:0_16:0). These phospholipids are key membrane constituents and signaling molecules; their observed alteration is consistent with adaptive membrane remodeling in response to the dietary energy shift [46], though this interpretation remains speculative. Furthermore, PC(16:0_16:0) was jointly enriched in both linoleic and α-linolenic acid metabolism, suggesting an active role in regulating unsaturated fatty acid fluxes.

In the 67BS vs. Control comparison, β-alanine was upregulated and enriched across retrograde endocannabinoid signaling, glycerophospholipid metabolism, and linoleic acid metabolism. β-alanine is a crucial precursor for pantothenate synthesis, which in turn is an essential component of coenzyme A (CoA). CoA directly activates and regulates lipid metabolism, including β-oxidation and de novo SFA synthesis [47]. Thus, the upregulation of β-alanine could be associated with enhanced CoA biosynthesis and altered fatty acid oxidation, but functional validation is required to confirm this. Concurrently, the downregulation of γ-glutamylcysteine, which serves as a precursor for glutathione synthesis, first appeared in the 67BS group, suggesting an early sign of altered tissue antioxidant status.

In contrast, the complete substitution (BS) group exhibited the most extensive metabolomic shift, characterized by the marked downregulation of glycine and related metabolites involved in glutathione metabolism, D-amino acid metabolism, and lysine degradation. Glycine is a vital substrate for glutathione synthesis. Its decline, coupled with the reduction in γ-glutamylcysteine and L-pyroglutamic acid, is consistent with a compromised antioxidant defense system in the muscle tissue [48]. This specific metabolic signature aligns with incipient oxidative stress in response to the all-barley diet; however, a direct mechanistic link to the observed decline in meat pH and altered color cannot be established from the present data. Additionally, the downregulation of 2-aminoadipic acid and N-α-acetyl-L-ornithine points to disruptions in lysine and arginine metabolism, which could negatively impact protein turnover and muscle growth [49]. The enrichment of the glycine, serine, and threonine metabolism, as well as the aminoacyl-tRNA biosynthesis pathways in the BS group, further corroborates a scenario of disrupted amino acid homeostasis, which may ultimately impair efficient muscle protein deposition.

### 4.6. Serum Bile Acid Parameters

The involvement of bile acid metabolism in diet–host interactions has gained increasing scientific attention. In this study, the 33BS group exhibited elevated serum concentrations of total bile acids (TBA), FGF-19, and CYP27A1 at day 30. FGF-19 is a critical endocrine signal released from the gut in response to bile acid absorption, acting to regulate hepatic bile acid synthesis [50]. The upregulation of CYP27A1, which represents a key enzyme in the alternative bile acid synthesis pathway, together with increased TBA, is consistent with a modulation of bile acid production and enterohepatic circulation by moderate barley inclusion. Because bile acids are essential for dietary lipid emulsification and act as potent metabolic regulators, this systemic response may be associated with the improved growth performance observed [51]. Concurrently, the decreased G-6-Pase levels observed in the 33BS and 67BS groups imply a potential downregulation of hepatic gluconeogenesis. This pattern aligns with the expected adaptive response to increased propionate supply and altered hormonal signaling, which would favor energy allocation toward tissue growth rather than hepatic glucose production; however, direct evidence for altered gluconeogenic flux is lacking in the present study.

### 4.7. Optimal Barley Inclusion Level and Multi-Tissue Integration

From a holistic perspective, the 33BS treatment emerged as the most advantageous dietary strategy. It consistently improved growth performance, enhanced ruminal propionate production, enriched the critical fiber-degrading bacterium Prevotella, and positively modulated systemic bile acid signaling, all without compromising rumen health or microbial diversity. While the 67BS level also offered improvements in growth and carcass traits, the significantly elevated ruminal ammonia and the trend toward reduced microbial evenness hint that it represents a marginal physiological optimum. Interestingly, the 67BS group exhibited the highest muscle levels of C4:0, C6:0, and C18:2t6, whereas the 33BS group excelled in enriching the essential fatty acid C18:3n-3. The all-barley diet (BS), despite not significantly impairing macroscopic growth, induced a profound loss of ruminal microbial diversity, triggered extensive muscle metabolic reprogramming indicative of oxidative stress, and reduced ultimate meat pH, all of which could severely compromise meat quality under commercial conditions. Therefore, replacing approximately one-third of dietary corn starch with barley starch is recommended for Arbas White Cashmere goats to maximize production performance while maintaining rumen homeostasis and desirable meat quality characteristics.

### 4.8. Limitations and Future Perspectives

Several limitations should be noted. First, the small sample size for omics analyses (n = 3 per group) and the short-term, single-breed design limit statistical power and generalizability; larger, long-term trials across multiple production cycles are needed. Second, the untargeted metabolomic and 16S rRNA gene-based functional predictions are correlative and rely on reference genomes; targeted quantitative metabolomics, isotope-tracing, and shotgun metagenomics are required to validate the proposed mechanisms. Third, the absence of direct measurements of antioxidant enzyme activities, oxidative damage markers, retail display stability, and consumer sensory evaluation constrains both mechanistic interpretation and practical application of the observed meat quality changes.

## 5. Conclusions

In summary, this integrated multi-omics study demonstrates that moderate dietary barley substitution, specifically at the 33% replacement level of corn starch, improves growth performance and carcass quality in cashmere goats. This improvement is underpinned by favorable shifts in rumen fermentation and a highly adaptive, moderate restructuring of the ruminal microbiota. Conversely, excessive replacement (100% barley starch) drastically reduced microbial diversity and triggered muscle metabolic alterations indicative of oxidative imbalance, offering no further growth advantages. The 33% barley inclusion level appears to balance productivity, rumen health, and meat quality, and these effects are associated with the enrichment of *Prevotella*, increased propionate production, and altered systemic bile acid metabolism. These findings provide a robust theoretical foundation for precision grain formulation in intensive cashmere goat production systems.

## Figures and Tables

**Table 1:** Basal diet and nutrient levels (air-dry basis, %).

Ingredient	CS	33BS	67BS	100BS
Corn stover	35.00	35.00	35.00	35.00
Corn	35.00	25.00	13.50	0.00
Barley	0.00	12.30	27.40	44.00
Wheat bran	7.00	6.70	4.60	2.00
Corn germ meal	6.00	3.00	2.00	2.00
Cottonseed meal	3.00	4.00	2.00	2.00
Soybean meal	8.00	8.00	9.50	9.00
Molasses	4.00	4.00	4.00	4.00
Dicalcium phosphate	0.20	0.20	0.20	0.20
Limestone	1.00	1.00	1.00	1.00
Salt	0.30	0.30	0.30	0.30
Premix ^1^	0.50	0.50	0.50	0.50
Total	100.00	100.00	100.00	100.00
Nutrient levels ^2^				
Metabolizable energy ^3^, ME (MJ/kg DM)	9.55	9.44	9.36	9.24
Dry matter, DM (%)	89.67	87.91	87.89	87.93
Crude protein, CP (% DM)	12.04	12.11	12.10	11.89
Calcium, Ca (% DM)	0.67	0.66	0.64	0.63
Phosphorus, P (% DM)	0.39	0.37	0.35	0.34
Neutral detergent fiber, NDF (g/kg DM)	321.71	313.92	305.98	302.20
Non-fiber carbohydrates, NFC (g/kg DM)	379.72	381.66	394.35	406.11
Barley starch: Corn starch ratio	0.00	33.00	67.00	100.00
Total starch (% of diet DM)	23.93	23.93	24.18	23.99

^1^ Provided per kg of premix: vitamin A, 1,000,000 IU; vitamin D_3_, 400,000 IU; vitamin E, 4500 IU; vitamin K_3_, 320 mg; vitamin B_1_, 60 mg; vitamin B_2_, 1600 mg; vitamin B_6_, 170 mg; nicotinic acid, 4.0 g; D-pantothenic acid, 3.2 g; VB_12_, 5 mg; biotin, 25 mg; folic acid, 280 mg; Fe, 7.5 g; Cu, 1.5 g; Zn, 9.5 g; Mn, 5.5 g; I, 55 mg; Se, 55 mg; Co, 45 mg. ^2^ All nutrient levels are measured values unless otherwise indicated. ^3^ Metabolizable energy was a calculated value. The four diets were formulated to maintain similar total dietary starch concentrations while varying the proportion of starch derived from barley relative to corn; the barley starch proportion is expressed as a percentage of total starch for clarity. CS, corn–soybean meal basal diet (100% corn starch); 33BS, 33% barley starch + 67% corn starch; 67BS, 67% barley starch + 33% corn starch; 100BS, 100% barley starch.

**Table 2:** Effects of barley replacement on growth performance of cashmere goats.

Item	CS	33BS	67BS	BS	SEM	*p*-Value
Initial body weight, Initial BW (kg)	21.24	22.80	22.36	22.96	1.195	0.738
Final body weight, Final BW (kg)	25.45 ^b^	29.82 ^a^	28.84 ^a^	28.00 ^ab^	1.019	0.039
Average daily feed intake, ADFI (g/d)	1111.94 ^b^	1343.00 ^a^	1253.66 ^ab^	1226.11 ^ab^	53.748	0.048
Average daily gain, ADG (g/d)	100.00 ^b^	122.66 ^a^	123.55 ^a^	111.85 ^ab^	6.296	0.041

Means within a row with different superscripts differ significantly (*p* < 0.05). CS, corn–soybean meal basal diet (0% barley); 33BS, 33% barley starch replacement; 67BS, 67% barley starch replacement; BS, 100% barley starch replacement. SEM, standard error of the mean. Means within a row without a common superscript letter differ significantly (*p* < 0.05).

**Table 3:** Effects of barley replacement on rumen fermentation parameters of cashmere goats.

Item	CS	33BS	67BS	BS	SEM	*p*-Value
Ammonia nitrogen (mg/dL)	8.95 ^b^	8.55 ^b^	16.22 ^a^	10.77 ^b^	1.437	0.019
pH	5.95 ^a^	5.42 ^b^	5.14 ^c^	5.45 ^b^	0.066	<0.001
Acetate (ng/μL)	28.49	38.51	38.85	33.20	5.472	0.524
Propionate (ng/μL)	10.56 ^b^	19.43 ^a^	13.42 ^b^	22.51 ^a^	1.369	0.001
Isobutyrate (ng/μL)	0.49	0.32	0.78	0.32	0.122	0.085
Butyrate (ng/μL)	5.87	6.20	10.07	6.38	1.727	0.340
Isovalerate (ng/μL)	0.55	0.22	0.45	0.38	0.110	0.281
Valerate (ng/μL)	0.82	1.39	0.96	0.89	0.220	0.320

CS, corn–soybean meal basal diet (0% barley); 33BS, 33% barley starch replacement; 67BS, 67% barley starch replacement; BS, 100% barley starch replacement. Means within a row without a common superscript letter differ significantly (*p* < 0.05).

**Table 4:** Effects of barley replacement on carcass performance of cashmere goats.

Item	CS	33BS	67BS	BS	SEM	*p*-Value
Carcass weight (kg)	10.66 ^c^	13.47 ^a^	13.17 ^ab^	11.43 ^bc^	0.549	0.018
Loin eye area (cm^2^)	12.01	13.43	16.32	7.84	1.936	0.077
GR value	15.67	15.75	10.8	15	2.841	0.583
Carcass length (cm)	60.02	69.17	62.67	64.67	2.609	0.177
Carcass anterior width (cm)	28.33	29.33	28.50	28.02	0.402	0.191
Carcass posterior width (cm)	19.33 ^c^	21.67 ^ab^	22.00 ^a^	19.67 ^bc^	0.629	0.036
Carcass depth (cm)	4.27	5.57	5.79	4.17	0.497	0.099

CS, corn–soybean meal basal diet (0% barley); 33BS, 33% barley starch replacement; 67BS, 67% barley starch replacement; BS, 100% barley starch replacement. Means within a row without a common superscript letter differ significantly (*p* < 0.05).

**Table 5:** Effects of barley replacement on meat quality of cashmere goats.

Item	CS	33BS	67BS	BS	SEM	*p*-Value
pH	6.24 ^a^	6.24 ^a^	6.27 ^a^	6.00 ^b^	0.073	0.049
Lightness (L*)	42.77 ^a^	37.75 ^b^	37.14 ^b^	38.99 ^b^	1.049	0.003
Redness (a*)	10.20 ^b^	11.91 ^a^	11.92 ^a^	12.09 ^a^	0.354	0.002
Yellowness (b*)	8.45	9.55	9.80	9.29	0.760	0.623

CS, corn–soybean meal basal diet (0% barley); 33BS, 33% barley starch replacement; 67BS, 67% barley starch replacement; BS, 100% barley starch replacement. Means within a row without a common superscript letter differ significantly (*p* < 0.05).

**Table 6:** Effects of barley replacement on nutrient composition of *Longissimus dorsi* in cashmere goats.

Item	CS	33BS	67BS	BS	SEM	*p*-Value
Dry matter (%)	27.05	27.08	24.38	27.41	0.017	0.590
Crude protein (% DM)	70.28	78.26	82.14	78.78	3.483	0.181
Crude fat (% DM)	22.95	19.04	10.29	16.93	0.034	0.160

CS, corn–soybean meal basal diet (0% barley); 33BS, 33% barley starch replacement; 67BS, 67% barley starch replacement; BS, 100% barley starch replacement.

**Table 7:** Effects of barley replacement on serum biochemical parameters of cashmere goats at day 30.

Item	CS	33BS	67BS	BS	SEM	*p*-Value
GLU (umol/L)	3.33	3.33	3.46	3.66	0.174	0.522
TBA (μmol/L)	62.16 ^b^	139.29 ^a^	104.57 ^ab^	70.32 ^b^	19.941	0.048
FGF15 (ng/L)	678.28	575.13	552.55	643.64	67.607	0.530
FGF-19 (pg/mL)	946.41 ^b^	1030.14 ^a^	988.78 ^ab^	903.35 ^b^	33.805	0.034
FGF-21 (ng/L)	925.01	907.49	966.49	866.16	98.789	0.911
CYP7A1 (ng/L)	290.82	302.23	354.82	313.64	32.117	0.533
CYP27A1 (ng/L)	176.32 ^b^	210.55^a^	176.49 ^b^	195.98 ^ab^	9.231	0.044
BSH (ng/L)	314.89	323.36	297.15	320.36	19.995	0.794
FBP1 (ng/L)	204.20	213.04	193.01	210.50	18.095	0.865
PEPCK (ng/L)	143.94	154.47	153.89	142.95	13.305	0.883
G-6-Pase (ng/L)	103.39 ^a^	83.79 ^b^	82.62 ^b^	94.52 ^ab^	5.474	0.045
CYP7B1 (pg/mL)	123.29	115.69	128.96	120.13	6.259	0.511
GLP-1 (pmol/L)	12.48	11.72	13.48	13.54	0.801	0.343
Insulin (μIU/mL)	13.32	17.86	14.29	17.43	1.514	0.117

CS, corn–soybean meal basal diet (0% barley); 33BS, 33% barley starch replacement; 67BS, 67% barley starch replacement; BS, 100% barley starch replacement. Means within a row without a common superscript letter differ significantly (*p* < 0.05).

**Table 8:** Effects of barley replacement on fatty acid composition (% of total fatty acids) of *Longissimus dorsi* in cashmere goats.

Item	CS	33BS	67BS	BS	SEM	*p*-Value
*Longissimus dorsi*						
C4:0	0.33 ^b^	0.19 ^b^	0.86 ^a^	0.03 ^b^	0.145	0.018
C6:0	0.02 ^b^	0.03 ^b^	0.09 ^a^	0.01 ^b^	0.011	0.005
C8:0	0.21	0.21	0.29	0.17	0.083	0.765
C10:0	0.04	0.07	0.05	0.03	0.032	0.837
C12:0	0.03	0.08	0.08	0.07	0.035	0.678
C13:0	0.01	0.02	0.02	0.02	0.007	0.482
C14:0	1.38	1.91	1.7	1.87	0.331	0.674
C15:0	0.2	0.26	0.28	0.28	0.050	0.646
C16:0	21.92	16.1	8.02	16.59	6.811	0.573
C17:0	0.78	0.9	0.03	0.02	0.293	0.122
C18:0	18.77 ^a^	15.93 ^ab^	14.08 ^b^	16.45 ^ab^	1.735	0.041
C20:0	0.01	0.02	0.02	0.01	0.011	0.837
C21:0	0.02	0.01	0.02	0.01	0.004	0.297
C22:0	0.01	0.01	0.02	0.02	0.004	0.492
C23:0	2.65	3.49	4.68	3.32	1.585	0.836
C24:0	0.32	0.32	0.41	0.44	0.121	0.804
C14:1	0.06	0.12	0.11	0.09	0.018	0.354
C15:1	7.27	7.18	15.39	7.71	6.527	0.470
C16:1	0.03	0.04	0.06	0.05	0.013	0.391
C17:1	0.05	0.05	0.04	0.08	0.019	0.454
C18:1t9	0.77	0.54	0.62	0.72	0.194	0.829
C18:1c9	50.79	50.14	49.45	47.65	2.072	0.739
C20:1	0.29	0.32	0.38	0.38	0.067	0.701
C22:1	0.04	0.06	0.06	0.08	0.026	0.772
C24:1	0.01 ^b^	0.05 ^b^	0.63 ^a^	0.16 ^b^	0.088	0.003
C18:2t6	0.11 ^b^	0.12 ^b^	0.16 ^a^	0.11 ^b^	0.009	0.025
C18:2c6	0.14	0.83	0.19	0.16	0.312	0.393
C18:3n6	0.03	0.03	0.04	0.04	0.008	0.776
C20:2n6	0.02	0.03	0.03	0.03	0.005	0.356
C20:3n6	0.18	0.22	0.27	0.25	0.067	0.801
C20:4n6	0.03	0.03	0.03	0.03	0.009	0.996
C18:3n3	0.12 ^c^	0.20 ^a^	0.17 ^b^	0.13 ^c^	0.016	0.001
C20:3n3	0.01	0.02	0.02	0.03	0.064	0.449
C20:5n3	0.04	0.03	0.03	0.03	0.014	0.911
C22:6n3	0.42 ^b^	0.69 ^ab^	0.80 ^a^	1.00 ^a^	0.165	0.015
SFA	46.71	39.54	31.79	40.18	5.599	0.374
UFA	53.29	60.46	68.21	59.82	5.599	0.374
PUFA	1.28	2.00	1.72	2.89	0.739	0.503
MUFA	52.02	51.28	51.11	49.22	2.014	0.79
n-3 PUFA	0.76	0.74	1.02	2.27	0.701	0.411
n-6 PUFA	0.51	1.26	0.72	0.62	0.281	0.311
n-3 LCPUFA	0.62	0.57	0.85	2.14	0.708	0.407
n-6 LCPUFA	0.23	0.28	0.33	0.32	0.078	0.802
n-6/n-3	0.71	2.43	0.68	0.55	0.814	0.369
U/S	1.15	1.72	2.40	1.61	0.467	0.363
P/S	0.03	0.05	0.06	0.07	0.017	0.368

CS, corn–soybean meal basal diet (0% barley); 33BS, 33% barley starch replacement; 67BS, 67% barley starch replacement; BS, 100% barley starch replacement. Means within a row without a common superscript letter differ significantly (*p* < 0.05).

**Table 9:** Selected significantly enriched KEGG pathways of differential metabolites in the *Longissimus dorsi* between B1 and B2 cashmere goats (B2 vs. B1) ^1^.

Pathway	DAMs ^2^	*p*-Value	Up ^3^	Down ^4^
Retrograde endocannabinoid signaling	2	0.0105	PE(P-18:1/20:4)	
			PC(16:0/16:0)	
Autophagy—other	1	0.0209	PE(P-18:1/20:4)	
Pathogenic Escherichia coli infection	1	0.0209	PE(P-18:1/20:4)	
Biosynthesis of alkaloids derived from shikimate pathway	3	0.0227	Emetine	Alpha-d-glucose
			Shikimate	
Glycosylphosphatidylinositol (GPI) anchor biosynthesis	1	0.0415	PE(P-18:1/20:4)	
Autophagy—animal	1	0.0415	PE(P-18:1/20:4)	
Proximal tubule bicarbonate reclamation	1	0.0415		Alpha-d-glucose
Kaposi sarcoma-associated herpesvirus infection	1	0.0415	PE(P-18:1/20:4)	
Glycerophospholipid metabolism	2	0.0459	PE(P-18:1/20:4)	
			PC(16:0/16:0)	
Linoleic acid metabolism	1	0.0616	PC(16:0/16:0)	
Caffeine metabolism	1	0.0814	3-Methylxanthine	
alpha-Linolenic acid metabolism	1	0.1008	PC(16:0/16:0)	

^1^ B2 vs. B1, differential metabolites in the experimental group B2 compared with the control group B1; ^2^ DAMs, differentially accumulated metabolites; ^3^ Up, metabolites significantly upregulated in group B2; ^4^ Down, metabolites significantly downregulated in group B2.

**Table 10:** Selected significantly enriched KEGG pathways of differential metabolites in the *Longissimus dorsi* between B1 and B3 cashmere goats (B3 vs. B1) ^1^.

Pathway	DAMs ^2^	*p*-Value	Up ^3^	Down ^4^
Retrograde endocannabinoid signaling	3	0.0105	Beta-alanine	2′-deoxycytidine 5′-monophosphate
				Thymidine
Autophagy—other	1	0.0209		O-phosphoethanolamine
Pathogenic Escherichia coli infection	2	0.0209	PC(16:0/16:0)	O-phosphoethanolamine
Biosynthesis of alkaloids derived from shikimate pathway	1	0.0227	PC(16:0/16:0)	
Glycosylphosphatidylinositol (GPI) anchor biosynthesis	1	0.0415	PC(16:0/16:0)	
Autophagy—animal	1	0.0415	Guanidinoethyl sulfonate	
Proximal tubule bicarbonate reclamation	1	0.0415	Digalacturonic acid	
Kaposi sarcoma-associated herpesvirus infection	1	0.0415		N-.alpha.-acetyl-l-ornithine
Glycerophospholipid metabolism	1	0.0459	Beta-alanine	
Linoleic acid metabolism	1	0.0616	Beta-alanine	
Caffeine metabolism	1	0.0814		gamma.-glu-cys

^1^ B3 vs. B1, differential metabolites in the experimental group B3 compared with the control group B1; ^2^ DAMs, differentially accumulated metabolites; ^3^ Up, metabolites significantly upregulated in group B3; ^4^ Down, metabolites significantly downregulated in group B3.

**Table 11:** Selected significantly enriched KEGG pathways of differential metabolites in the *Longissimus dorsi* between B1 and B4 cashmere goats (B4 vs. B1) ^1^.

Pathway	DAMs ^2^	*p*-Value	Up ^3^	Down ^4^
Glutathione metabolism	3	0.0024		gamma.-glu-cys
				Glycine
				L-pyroglutamic acid
D-Amino acid metabolism	3	0.0066	cis-4-Hydroxy-D-proline	D-glutamine
				Glycine
Lysine degradation	3	0.0086	Glutaric acid	2-aminoadipic acid
				Glycine
Microbial metabolism in diverse environments	8	0.0246	4-chlorophenoxyacetic acid	3-hydroxyphenylacetic acid
			Gallic acid	Glycine
			Glutaric acid	P-toluenesulfonic acid
			Phosphoserine	Resorcinol
Aminoacyl-tRNA biosynthesis	2	0.0342	Phosphoserine	Glycine
Biosynthesis of amino acids	4	0.0506	Phosphoserine	2-aminoadipic acid
				Glycine
				N-.alpha.-acetyl-l-ornithine
Penicillin and cephalosporin biosynthesis	1	0.0753		2-aminoadipic acid
Glycosylphosphatidylinositol (GPI) anchor biosynthesis	1	0.0753		O-phosphoethanolamine
Pyrimidine metabolism	3	0.0937	Deoxythymidine triphosphate	2′-deoxycytidine 5′-monophosphate
			Uridine 5′-monophosphate	
Synaptic vesicle cycle	1	0.1453		Glycine
Glycine, serine and threonine metabolism	2	0.1622	Phosphoserine	Glycine
2-Oxocarboxylic acid metabolism	2	0.1622		2-aminoadipic acid
				N-.alpha.-acetyl-l-ornithine

^1^ B4 vs. B1, differential metabolites in the experimental group B4 compared with the control group B1; ^2^ DAMs, differentially accumulated metabolites; ^3^ Up, metabolites significantly upregulated in group B4; ^4^ Down, metabolites significantly downregulated in group B4.

## Data Availability

The original contributions presented in this study are included in the article. Further inquiries can be directed to the corresponding author.
